# Impact of Repeated Exposure to Polarized Health-Related News on Explicit and Implicit Attitudes Toward Dietary Supplements: Online Experimental Study

**DOI:** 10.2196/88632

**Published:** 2026-07-27

**Authors:** Eugen-Călin Secară, Nicolae-Adrian Opre

**Affiliations:** 1 Department of Psychology, Faculty of Psychology and Educational Sciences, Universitatea Babeș-Bolyai, Mihail Kogălniceanu 1, Cluj-Napoca, Cluj, 400347, Romania, 40 264405337

**Keywords:** illusory truth effect, mere exposure effect, implicit attitudes, explicit attitudes, health-related content, polarization, repeated exposure, misinformation, public health, health information, echo chamber

## Abstract

**Background:**

Repetition is a central feature of digital news consumption, where engagement-driven algorithms often expose users to similar health-related content. Prior research suggests that repeated exposure can influence perceived truth and evaluation, but most studies use brief or decontextualized stimuli and have rarely distinguished between explicit and implicit attitudes. Little is known about how repeated exposure to full-length, polarized health news shapes explicit and implicit attitudes, particularly toward familiar products such as dietary supplements.

**Objective:**

This study aims to examine whether 2 weeks of exposure to positively, negatively, or mixed-valence health news articles would alter explicit and implicit attitudes toward dietary supplements, and whether engagement mediated these effects or initial attitudes moderated them.

**Methods:**

In a preregistered 4 (group: PRO, CON, MIX, and control) × 3 (time: T0, T1, and T2) mixed experimental study, 228 participants (174 women, PRO: n=68, CON: n=51, MIX: n=52, control: n=57; mean age 2.81, SD 4.88 years) were randomly assigned to receive one full-length article per day for 2 weeks. Articles presented positive (PRO), negative (CON), mixed (MIX), or neutral space-related information (control). Explicit attitudes toward dietary supplements (perceived efficiency, harmfulness, and willingness to recommend) were assessed with visual analog scales, and implicit attitudes with an Implicit Association Test at baseline, 1 week, and 2 weeks. Mixed analysis of covariances (ANCOVAs) controlled for self-reported exposure to health-related news. Mediation, moderation, and moderated mediation analyses examined whether time spent reading and baseline attitudes influenced change.

**Results:**

The change in implicit attitudes was not significant (*F*_6, 428_=1.90; *P*=.08). In contrast, explicit attitudes showed a significant group × time interaction (*F*_5.04, 428_=14.34; *P*<.001). Explicit attitudes became more favorable in the PRO group (M_Δ_=14.86, SE=5.93, *d*=0.30; *P*=.02) and less in the CON (M*_Δ_*=–53.42, SE=7.35, *d*=1.02; *P*<.001) and MIX groups (M_Δ_=–24.56, SE=6.77, *d*=0.50; *P*=.001). At T2, the CON group reported lower explicit attitudes than the control group (M_Δ_=–32.84, SE=1.75, *d*=0.32; *P*=.02), and the PRO group scored higher than the CON and MIX groups (M_Δ_=52.08, *d*=0.52; *P*<.001 and M_Δ_=27.07, *d*=0.29; *P*=.03). Exploratory item analyses showed large effects of negative exposure on perceived harmfulness and recommendability. No mediation by reading time and no moderation by baseline attitudes were supported.

**Conclusions:**

Repeated exposure to polarized health-related news shifted explicit but not implicit attitudes toward dietary supplements. Negative content exerted the strongest influence. Engagement and prior attitudes did not meaningfully shape these outcomes. These findings suggest that even brief, routine exposure to polarized health information may accumulate into explicit evaluative change, underscoring the importance of balanced digital news environments.

## Introduction

### Background

Exposure to online information is increasingly shaped by algorithms designed to optimize user engagement. Combined with the decline of traditional news sources [1] and the growing prevalence of paid promotional content within news feeds [2], this shift has raised concerns about exposure to information that aligns with users’ existing beliefs while limiting contact with opposing viewpoints, a phenomenon commonly described as echo chambers [3,4]. Although recent evidence suggests that the polarizing effects of political echo chambers are limited for most users [5-8], health-related content (HRC) has exhibited a marked informational divide, particularly during the COVID-19 pandemic [9], where automated distribution mechanisms such as bots contributed to the spread of unreliable information [10].

Unlike political content, HRC is frequently linked to direct commercial interests, often promoting specific products or brands. Because sensationalist content generates greater engagement than balanced reporting [11], commercial incentives may encourage polarized framing and selective presentation of evidence. Beyond restricting informational diversity, health-related echo chambers repeatedly expose users to similar information, engaging cognitive processes through which repetition can alter evaluations regardless of accuracy [12,13].

### Repetition and Evaluation

The mere exposure effect describes the tendency for repeated encounters with a stimulus to increase positive affect and perceived familiarity even in the absence of conscious recognition or evaluative intent [12], an effect observed across a wide range of stimuli including words, symbols, and faces [14]. In online health communication, repeated exposure to a claim, product, or figure may therefore enhance likability, irrespective of its accuracy.

Closely related is the illusory truth effect, whereby repeated statements are judged as more truthful than novel ones [13,15]. This effect has been demonstrated across variations in plausibility [16], cognitive ability, and cognitive style [17], and persists even when information is explicitly qualified or labeled as uncertain, improbable, or advertising [18,19]. Initial repetitions produce the greatest increases in truth judgments [20] and spaced repetitions exert stronger effects [21,22]. Studies examining HRC have demonstrated increased belief in both true and false repeated claims about tobacco [23] and COVID-19 [24]. Repetition increases processing fluency, the ease with which information is perceived and retrieved, which is then misattributed to truthfulness rather than familiarity itself [25,26].

In algorithmically mediated environments, where users are repeatedly exposed to similar health-related information, such fluency-driven processes may systematically influence evaluations and beliefs.

### Explicit and Implicit Attitudes

Human behavior reflects the joint influence of explicit and implicit attitudes, 2 evaluative systems that differ in awareness, control, and their impact on action. Explicit attitudes are consciously accessible evaluations that can be reported and reflected upon, whereas implicit attitudes are automatic associations that may operate outside conscious awareness and diverge from self-reported beliefs, influencing spontaneous or habitual behavior [27-29].

Explicit attitudes predict deliberate and controlled actions, such as making health decisions after reflection or expressing stated opinions [30]. Implicit attitudes predict spontaneous or nonverbal behaviors, such as avoidance, facial expression, or tone, particularly under conditions of limited time or cognitive resources [31]. The interaction between the 2 systems can produce inconsistency between what people *say* and what they *do*, a discrepancy relevant for understanding reactions to polarized health information, where explicit belief change may not be reflected in behavioral tendencies.

Within polarized information environments, repeated exposure can be expected to affect explicit attitudes through fluency-driven increases in perceived truth (ie, the illusory truth effect) and to influence implicit attitudes through consistent evaluative pairings that strengthen affective associations [32] or through the presentation of crucial information which leads to the reinterpretation of previously held attitudes [33-35].

### The Current Research

The aim of this study was to investigate how repeated exposure to polarized health information influences explicit and implicit attitudes toward dietary supplements. Prior studies on the illusory truth effect and evaluative conditioning have typically relied on isolated statements or short stimuli, limiting ecological validity. To better approximate real-world information exposure, this study examined the effects of repeated exposure to full-length online articles containing positive, negative, or neutral information.

Repeated exposure to congruent information was expected to shift attitudes in the direction of the presented valence. Specifically, positive exposure was expected to increase favorable implicit (H1a) and explicit (H1b) attitudes, whereas negative exposure was expected to decrease favorable implicit (H2a) and explicit (H2b) attitudes. Exposure to both positive and negative information was expected to produce changes in attitudes over time (H3a, H3b). Comparisons with a neutral control condition were expected to clarify whether any observed changes reflected informational valence rather than repeated measurement or the passage of time (H4-H6).

Beyond these primary effects, the study examined whether baseline attitudes moderated attitude change (H7), anticipating that participants holding more positive initial views of dietary supplements would show smaller changes when exposed to positive information but larger negative shifts in the negative exposure condition, based on how diagnostic the nature of the presented information is with regard to previously held beliefs. Moreover, the relationship between informational valence and attitude change was expected to be mediated by engagement, such that greater time spent reading articles would predict stronger attitude change (H8). Finally, this mediation was hypothesized to be moderated by initial attitudes, reflecting the interaction between prior beliefs and the effects of repeated exposure (H9).

## Methods

### Design and Procedure

**Figure 1.**
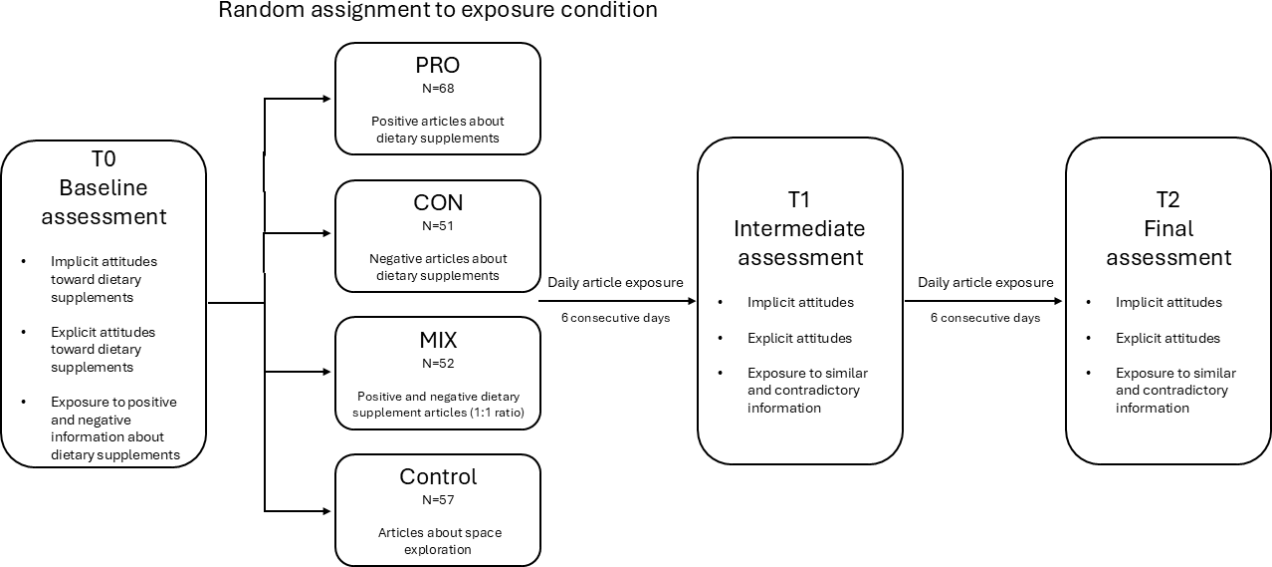
Study design*.*

The study used a 4×3 mixed experimental design. The between-subjects factor was group, defined by the type of articles participants read (PRO: positive information about dietary supplements; CON: negative information about dietary supplements; MIX: both positive and negative information regarding dietary supplements presented across different articles; or Control: neutral content about space exploration, unrelated to health), and the within-subjects factor was Time, with assessments at baseline (T0), 1 week (T1), and 2 weeks (T2). Participants were randomized with replacement by the Gorilla online experimental platform [36] into 1 of the 4 groups before exposure began. An overview of the study design is presented in Figure 1.

Participants accessed the study via a link to the Gorilla platform. They were presented with a cover story stating that the purpose of the project was to assess personal characteristics of readers who enjoy different types of online articles. After providing informed consent, participants completed the initial assessment (T0) and were randomized into 1 of the 4 groups, being presented with the first article of their assigned condition. After reading, they rated the article on a 4-star scale and were informed that their next article would be available the following day.

Each participant then received 1 article per day for 6 consecutive days, followed on the seventh day by the second assessment (T1). The procedure was repeated for a second cycle of 6 daily articles, after which participants completed the final assessment (T2). The platform recorded time spent reading each article (in seconds). Each assessment included measures of explicit attitudes, implicit attitudes, and frequency of online exposure to health information that either aligned with or contradicted the study content.

The article stimuli were developed in November 2022. To approximate the type of information users may encounter when searching for dietary supplement-related content online, the first 20 Google results pages were screened for a range of commonly searched dietary supplement topics spanning general supplement use and several health-related domains. The complete list of search terms is provided in Table S1 of Multimedia Appendix 1.

Candidate articles were selected to represent recurring perspectives commonly encountered in online discussions of dietary supplements. Attention was paid to recurring themes related to nutrition, natural and traditional remedies, mechanisms and effectiveness, and safety considerations. Within each perspective, preference was given to articles that presented more developed arguments, referred to supporting evidence or expert recommendations where available, and remained understandable to a general audience. To maintain consistency across stimuli, selected articles included a short introduction and favored repeated references to supplement-related terms such as “vitamins,” “minerals,” “nutrients,” and “amino acids.”

Information from selected articles was reorganized and edited to create stimuli of comparable length (approximately 450‐500 words). When source articles contained both positive and negative information, content was separated by valence to maintain experimental control while preserving the original thematic content. For example, one negative stimulus (C1) was constructed from a Buiced article [37], retaining only information concerning potential side effects, limited premarket evidence requirements, and the distinction between naturalness and safety. This material was sufficient to reach the target length without supplementation from additional sources.

In contrast, one positive stimulus (P1) drew its introduction from a Nova Southeastern University article [38] and retained positively framed information concerning supplement effectiveness, safety, and marketplace oversight. Because this content did not reach the target length, additional material regarding rigorous state regulation of the dietary supplement marketplace from the Buiced article [37] was incorporated.

Using this procedure, 28 stimuli were initially developed and pretested (Table S1 in Multimedia Appendix 1). Of these, 24 stimuli were ultimately included in the study (12 PRO and 12 CON articles). Table 1 summarizes the topics and recurring themes represented in the final stimulus set, together with illustrative examples of how these themes were instantiated within each stimulus. Full stimuli are available in Multimedia Appendix 2.

**Table 1.** Recurring themes represented across dietary supplement topics and their instantiation within the article stimuli.

Theme	General supplement use	Sleep	Weight loss	Influenza	Mental health	Diabetes
Nutrition	P1^a^ (nutrient gaps), P2 (deficiencies), C1^b^ (diet first)	P4 (vitamin B6; magnesium)	P5 (diet support)	P8 (vitamin C, D), C8 (diet)	P10 (mood enhancer)	P11 (micronutrients), C12 (diet first)
Natural or traditional remedies	P2 (plant based), C2 (natural ≠ safe)	P3 (valerian; 5-HTP), P4 (lemon balm; GABA), C3 (sleep aids), C4 (valerian; chamomile)	P6 (green tea; chitosan), C6 (weight-loss botanicals)	P7 (ginger; turmeric), P8 (garlic; green tea), C7 (herbal supplements)	P9 (passionflower; ashwagandha), P10 (L-theanine; probiotics), C9 (kava; valerian), C10 (passionflower; St. John’s wort)	P12 (bitter melon; chromium), C11 (Ayurvedic products)
Mechanisms, evidence, safety	P1 (evidence), P2 (expert opinion), C1 (toxicity; interactions), C2 (adverse effects)	P3 (sleep quality), P4 (sleep regulation), C3 (side effects; interactions), C4 (efficacy concerns; interactions)	P5 (weight management), P6 (fat metabolism), C5 (limited efficacy; adverse effects), C6 (stimulant risks)	P7 (immune enhancement), P8 (viral inhibition), C7 (limited evidence; interactions), C8 (antioxidant risks)	P9 (anxiety reduction), P10 (mood regulation), C9 (side effects; interactions), C10 (limited evidence; risks)	P11 (glucose control), P12 (AMPK; glucose uptake), C11 (hypoglycemia risk), C12 (limited evidence; interactions)
Regulation, product quality	P1 (GMP standards), C1 (premarket exemption), C2 (quality variability)	C3 (unregulated products), C4 (ingredient variability)	C5 (fraudulent products), C6 (FDA^c^ bans)	C7 (quality variability)	C9 (quality concerns)	C11 (illegal products), C12 (marketing loopholes)

a P: positive article (PRO exposure group).

b C: negative article (CON exposure group).

c FDA: US Food and Drug Administration.

The MIX condition was intended to approximate exposure to competing health narratives encountered across different information sources, rather than balanced reporting within a single article. Due to a technical error in the platform, participants in the MIX group did not receive articles in the intended randomized order. Instead, they were systematically presented with a positive article followed by a negative one, such that the day immediately preceding each assessment (T1 and T2) always featured a negative (CON-type) article. This deviation was consistent across all MIX participants and was documented prior to data analysis.

During the initial visit to the platform, participants provided contact information that was used to remind them to engage daily with the study materials. They subsequently received reminders via email, SMS text message, and, when no response was recorded by the evening, by telephone. Because the platform could not advance without completing the daily task, participants who missed a day were automatically removed from the study and received no further notifications.

### Participants

Participants were recruited from the Babeș-Bolyai University student population, who were offered course credit in exchange for participation, and from the general Romanian population through social media posts and targeted online advertisements. Inclusion criteria required participants to be at least 18 years old and to have access to a keyboard-compatible device for completing the online tasks. A power analysis based on the procedures described by Preacher et al [39] indicated that a total sample of 200 participants (50 per group) would provide sufficient power to detect a medium-sized conditional indirect effect in moderated mediation models. Recruitment was planned to reach this target, and data collection concluded once it was achieved, with participants who had already begun the study allowed to complete their participation.

A total of 345 participants initially enrolled in the study. Of these, 117 dropped out during the study, resulting in a final sample of 228 participants. Demographic characteristics of the final sample are presented in Table 2. Participants were distributed across conditions as follows: PRO (n=68), CON (n=51), MIX (n=52), and Control (n=57).

**Table 2.** Demographic characteristics of the final sample.

Characteristics	Values
Age (years), mean (SD)	20.81 (4.88)
Gender, n (%)	
Women	174 (76.3)
Men	52 (22.8)
Other or prefer not to say	2 (0.9)
Qualifications, n (%)	
Primary education	1 (0.4)
Secondary education	169 (74.1)
Undergraduate degree	42 (18.4)
Graduate degree	16 (7)

### Measures

#### Implicit Attitudes

Implicit attitudes toward dietary supplements were assessed using a computerized Implicit Association Test (IAT, [40]) administered via the Gorilla online platform. The IAT measured the relative strength of automatic associations between dietary supplements and positive versus negative evaluative attributes.

Participants categorized stimuli into one of two target categories—dietary supplements (eg, “vitamins,” “minerals,” “nutrients”) and objects (eg, “trousers,” “chair,” “poster”)—and two attribute categories—positive (eg, “healthy,” “good,” “safe”) and negative (eg, “risky,” “bad,” “dangerous”). During the task, single words appeared sequentially in the center of the screen, and participants were instructed to classify each as quickly and accurately as possible using 2 response keys corresponding to the category pairings displayed on-screen. The task alternated between congruent blocks (eg, dietary supplements + positive and objects + negative) and incongruent blocks (eg, dietary supplements + negative and objects + positive). Reaction times and accuracy were recorded. Incorrect responses triggered an error message, and the trial continued only after the correct key was pressed, penalizing errors through longer response times [41].

An IAT D-score was computed for each participant at each time point (T0, T1, and T2) following standard scoring procedures [41]. The D-score was calculated by subtracting the mean reaction time for congruent blocks from that for incongruent blocks and dividing by the pooled standard deviation of all response latencies. Higher D-scores indicated more favorable implicit attitudes toward dietary supplements.

#### Explicit Attitudes

Explicit attitudes were measured using three visual analog scale (VAS) questions: “To what degree do you consider dietary supplements to be efficient?”; “To what degree do you consider dietary supplements to be harmful?” (reverse-scored); and “To what degree would you recommend dietary supplements to a loved one?” Scores were aggregated across items at each time point to yield an explicit attitude index, with higher values reflecting more favorable evaluations. Internal consistency was acceptable at every evaluation (Cronbach *α*=0.79, 0.75, and 0.76, respectively).

The frequency of exposure to health-related news and similar news was assessed using the following questions: “How often do you read health-related news?” (only at T0); “During the last week, how often have you encountered positive news articles related to dietary supplements?” (only at T0), “During the last week, how often have you encountered negative news articles related to dietary supplements?” (only at T0) “During the last week, how often have you encountered similar news articles to those presented on this platform?” (T1 and T2), “During the last week, how often have you encountered news articles that contradicted those presented on this platform?” (T1 and T2); and “Approximately how much time did you spend last week reading health-related news?.”

### Statistical Analysis

Data were analyzed using mixed analysis of covariance (ANCOVA) models with group as the between-subjects factor and time as the within-subjects factor. Dependent variables were explicit and implicit attitude scores. Covariates included time spent reading health-related news and self-reported exposure to health-related news on the topic of dietary supplements, assessing both similar and contradictory news.

Planned contrasts compared T2-T0 changes within the PRO, CON, and MIX groups and between-group differences at T2. One-tailed significance tests were used in accordance with our directional hypotheses (PRO and CON), and 2-tailed tests for the nondirectional hypothesis about the MIX group.

Moderation, mediation, and moderated mediation analyses were conducted using PROCESS [42]. These analyses tested whether baseline attitudes moderated the effect of group assignment on attitude change, whether time spent reading mediated this effect, and whether this mediation was further moderated by initial attitudes or exposure to similar news. Indirect effects were evaluated using bias-corrected bootstrapped confidence intervals (BootCI).

All data were analyzed using IBM SPSS 25.

### Ethical Considerations

The research was conducted within the framework of the author’s doctoral project at Babeș-Bolyai University, which received a doctoral scholarship from the Romanian Ministry of Education. At the time of doctoral enrollment (2019), ethical approval for the doctoral research project was granted through the institutional approval process rather than through separate approvals for individual studies. Consequently, the project is registered under reference number 14459/01.10.2019. The responsible ethics committee was the Research Ethics Subcommittee of Babeș-Bolyai University, Cluj-Napoca.

All participants provided informed consent prior to enrollment. Participants were compensated either with course credit (students) or entry into a lottery for one of ten €20 (US $21.65) prizes. Participants were debriefed about the true purpose of the study upon completion of data collection. Participant data were securely collected and stored on the Gorilla.sc research platform. Access to the research data was restricted to the research team, and all analyses and reporting were conducted using anonymized, aggregated data. Contact information was collected solely for the purpose of sending reminders during the 2-week study period and, where applicable, administering course credit or participation in the optional prize lottery. Research team members responsible for contacting participants had access only to participants’ participation status (ie, whether they had completed the current day's session) and not to their study responses. Following completion of the study, final debriefing, allocation of course credit, and administration of the optional lottery, all contact information was permanently deleted.

## Results

Descriptive statistics for the primary study variables are presented in Table 3. These include explicit and implicit attitudes toward dietary supplements, assessed at baseline (T0), 1 week (T1), and 2 weeks (T2), as well as a measure of participant engagement with the study materials, operationalized as the average time spent reading the articles.

**Table 3.** Descriptive statistics.

Variables	PRO (n=68), mean (SD)	CON (n=51), mean (SD)	MIX (n=52), mean (SD)	Control (n=57), mean (SD)
T0				
Implicit attitudes	−0.08 (0.42)	0 (0.48)	−0.12 (0.46)	−0.17 (0.43)
Explicit attitudes	190.21 (62.74)	202.25 (56.37)	200.17 (44.44)	203.60 (48.83)
Efficiency rating	61.99 (24.68)	66.86 (20.82)	67.79 (16.83)	67.18 (20.17)
Harmfulness rating	29 (19.87)	24.25 (18.10)	29.06 (20.62)	26.16 (17.44)
Recommendation willingness	56.22 (27.11)	58.65 (25.43)	60.44 (19.42)	61.58 (22.79)
Time spent reading study articles (s)	1164.82 (751.06)	1205.21 (1117.17)	1556.27 (106.60)	1399.18 (856.19)
T1				
Implicit attitudes	−0.05 (0.43)	−0.13 (0.47)	−0.30 (0.47)	−0.18 (0.45)
Explicit attitudes	201.31 (51.03)	150.80 (57.67)	184.92 (41.35)	188.12 (49.91)
Efficiency rating	67.93 (21.33)	49.82 (21.43)	64.29 (18.69)	62.72 (20.07)
Harmfulness rating	27.69 (20.65)	45.35 (22.19)	35.02 (18.06)	28.77 (18.48)
Recommendation willingness	60.07 (22.32)	45.33 (23.12)	54.65 (21.34)	53.18 (23.06)
T2				
Implicit attitudes	−0.15 (0.41)	−0.09 (0.39)	−0.23 (0.41)	−0.13 (0.40)
Explicit attitudes	206.15 (49.22)	148.06 (55.75)	176.40 (45.61)	187.54 (52.23)
Efficiency rating	68.94 (18.67)	47.37 (22.13)	61.58 (20.45)	60.65 (22.06)
Harmfulness rating	26.01 (18.36)	44.08 (20.93)	37.62 (21.13)	25.98 (16.56)
Recommendation willingness	62.22 (20.84)	43.76 (23.65)	51.44 (23.20)	51.88 (24.04)

Self-reported frequency of exposure to health-related news, time spent reading such news, and exposure to positive and negative articles about dietary supplements outside the experimental context were also assessed at each time point and included in the analyses as control variables. Full descriptive data for these control measures are available in Table S2 of Multimedia Appendix 1.

To examine changes in implicit attitudes, a 4×3 mixed-design ANCOVA was conducted on IAT *D*-scores across the 3 assessments, with group (PRO, CON, MIX, and Control) as the between-subjects factor and time (T0, T1, and T2) as the within-subjects factor. Time spent reading health-related news, frequency of exposure to health-related information (T0, T1, and T2), and time spent reading pro- and antisupplement articles (T0, T1, and T2) were included as covariates. The analysis revealed that the Group × Time interaction was not statistically significant *(F*_6, 428_=1.90, *η²ₚ*=0.03; *P*=.08). Given the absence of a significant interaction, no follow-up contrasts were performed. Hence, the collected data were insufficient to support H1a, H2a, H3a, H4a, H5a, and H6a.

**Figure 2.**
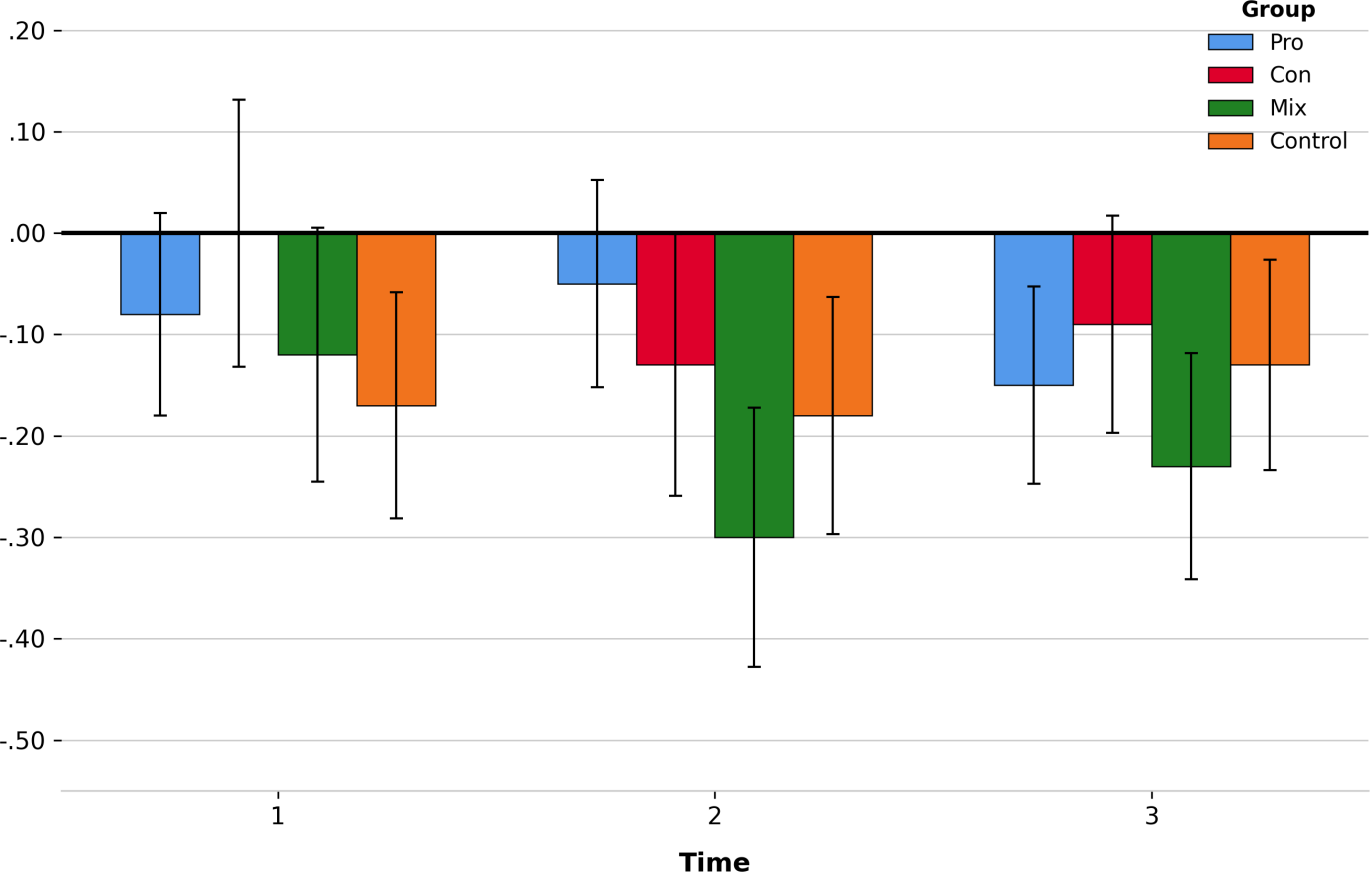
Changes in implicit attitudes toward dietary supplements across time as a function of exposure condition*.*

As illustrated in Figure 2, implicit attitudes remained relatively stable across time and exposure conditions.

To analyze changes in explicit attitudes toward dietary supplements, a 4×3 mixed-design ANCOVA was conducted on the composite explicit attitude scores, with group (PRO, CON, MIX, and control) as the between-subjects factor and time (T0, T1, and T2) as the within-subjects factor. Time spent reading health-related news, frequency of exposure to health-related information (T0, T1, and T2), and time spent reading pro- and anti-supplement articles (T0, T1, and T2) were included as covariates.

The analysis revealed a significant Group × Time interaction (*F*_5.04, 428_=14.34, *η²ₚ*=0.167; *P*<.001), showing that changes in explicit attitudes differed across exposure conditions.

**Figure 3.**
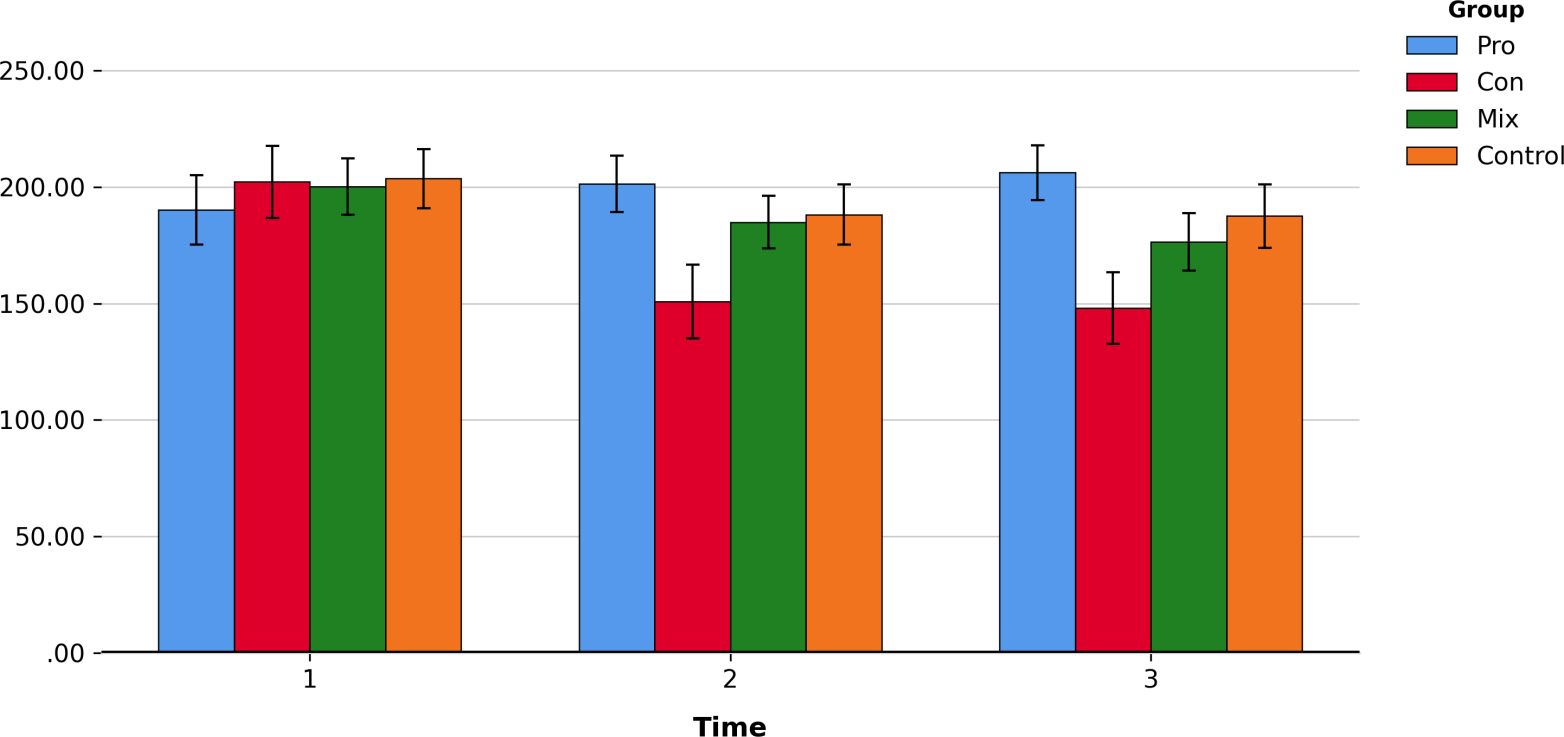
Changes in explicit attitudes toward dietary supplements across time as a function of exposure condition*.*

Preregistered within-group comparisons, illustrated in Figure 3, showed that participants in the PRO condition developed significantly more favorable explicit attitudes from baseline to the final assessment (MΔ=−14.86, SE=5.93, *d*=0.30; *P*_1-tailed_=.02). Participants in the CON condition showed the opposite pattern, reporting less favorable attitudes over time (M_Δ_=−53.42, SE=7.35, *d*=1.02; *P*_1-tailed_<.001). The MIX group exhibited a significant change consistent with the most recent exposure (ie, negative article, M_Δ_=−24.56, SE=6.77, *d*=0.50; *P*=.001), whereas the control group showed no significant change (M_Δ_=−14.74, SE=6.63, *d*=0.29; *P*=.08). H1b, H2b, and H3b are therefore supported by the collected data.

Between-group comparisons at the final assessment (T2) confirmed this pattern. The CON group reported significantly lower explicit attitudes than the control group (M_Δ_=−32.84, SE=10.75, *d*=0.32; *P*=.02). Explicit attitudes in the PRO group were not significantly different from the control group (M_Δ_=19.24, SE=9.29, *d*=0.20; *P*=.22) but were significantly higher than in the CON group (M_Δ_=52.08, SE=10.03, *d*=0.52; *P*<.001), and than in the MIX group (M_Δ_=27.07, SE=9.38, *d*=0.29; *P*=.03). No other between-group differences were significant. Data provided clear support for H5b and partial support for H4b.

Together, these results indicate that repeated exposure to polarized information altered explicit, but not implicit, attitudes toward dietary supplements, and that these changes occurred in the direction of the informational valence of the exposure.

In order to test whether baseline attitudes moderated the relationship between group assignment and attitude change, 2 multiple regression models were conducted. The model having implicit attitude change as criterion was statistically significant (*F*_11, 216_=16.80, *R^2^*=0.46; *P*<.001), but the only significant predictor was baseline implicit attitudes (*b*=–.84, *SE*=.11; *P*<.001). For the model having explicit attitude change as criterion, neither implicit (*F*_3, 216_=0.04, *ΔR^2^*=0.00; *P*=.99) nor explicit attitudes (*F*_3, 216_=1.94, *ΔR^2^*=0.02; *P*=.12) moderated the effect. Thus, the collected data were insufficient to support H7a and H7b.

Given that the mixed-design ANCOVA revealed no significant interaction between time and group for implicit attitudes and no consistent within-group changes, mediation analyses involving implicit attitudes as the outcome were not pursued. Therefore, H8a and H9a are not supported.

For explicit attitudes, the simple mediation model testing whether time spent reading mediated the relationship between exposure condition and explicit attitudes at T2 failed to meet the assumptions for mediation, as the path from group to reading time was nonsignificant (*F*_3, 224_=0.26, *R²*=0.03; *P*=.12). The collected data were not sufficient to support H8b.

To further examine whether engagement effects depended on participants’ initial evaluations, 2 preregistered moderated mediation models (PROCESS Model 7 [42]) were tested. When baseline implicit attitudes were included as a moderator of the Group to Reading (a) path, the model was still not significant (*F*_7, 220_=0.98, *R²*=0.03; *P*=.45). The analysis with baseline explicit attitudes as the moderator also yielded a nonsignificant model (*F*_7, 220_=1.06, *R²*=0.03; *P*=.39). Therefore, H9 is not supported by the collected data. A graphical representation of the tested moderated mediation models is provided in Figure S1 of Multimedia Appendix 1.

**Figure 4.**
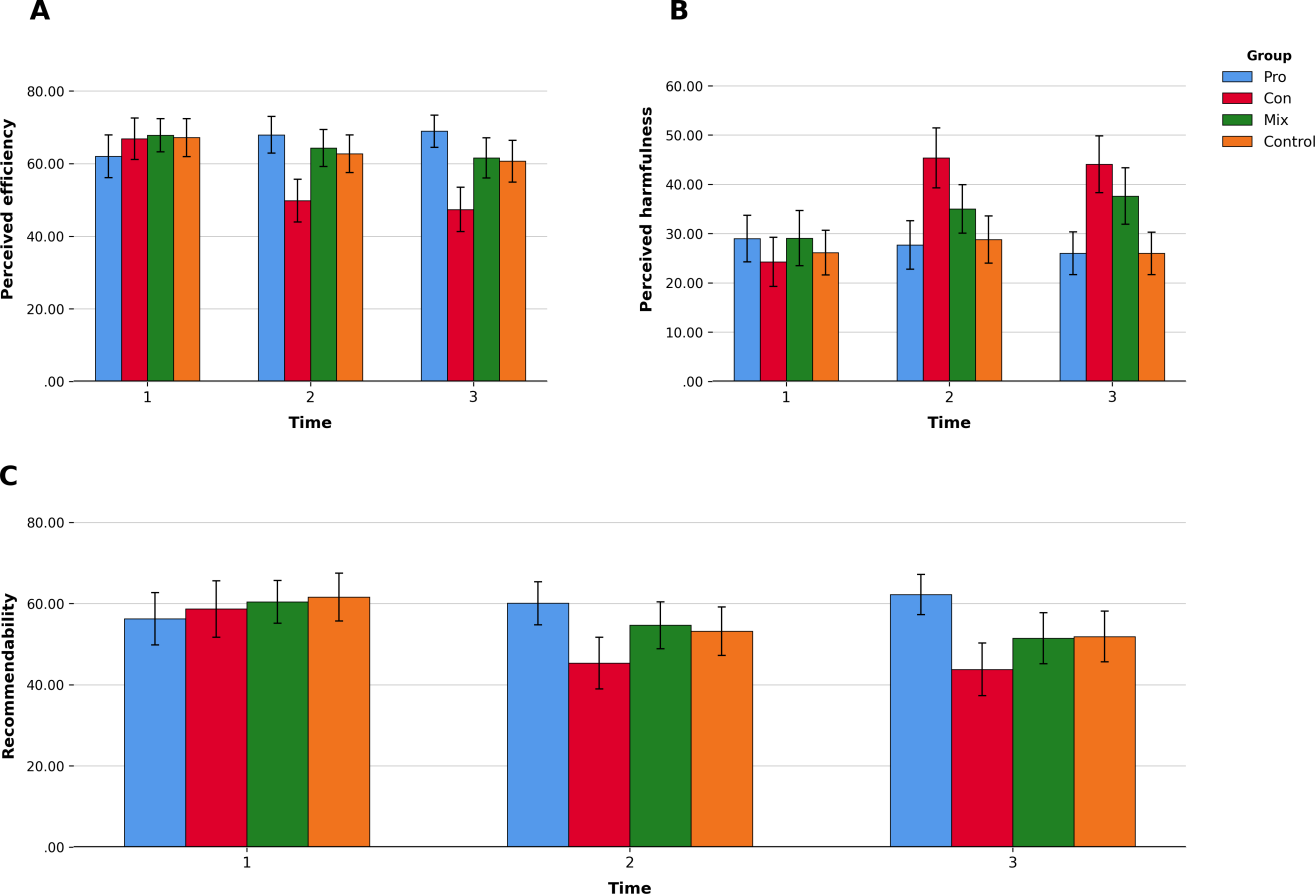
Exploratory analyses of the components of explicit attitudes toward dietary supplements. (A) perceived efficiency, (B) perceived harmfulness, and (C) recommendability.

To further examine the changes in explicit attitudes, the 3 items composing the composite score (ie, perceived efficiency, perceived harmfulness, and recommendability of dietary supplements) were analyzed separately using 4×3 mixed-design ANCOVAs with the same covariates as the main model. These analyses were treated as exploratory as they have not been preregistered. Figure 4 presents the changes in perceived efficiency, harmfulness, and recommendability across time and exposure conditions.

The multivariate test revealed a significant time × group interaction (*F*_18, 1344_=6.27, *η²_p_*=0.078; *P*<.001). At the univariate level, all 3 attitude items showed significant time × group interactions (all *Ps*<.001, *partial η²*s=0.09-0.14).

Perceived efficiency increased significantly in the PRO group from T0 to T2 (M_Δ_=6.96, *d*=0.42; *P*=.02) and decreased significantly in the CON group (M_Δ_=−19.49, *d*=0.83; *P*<.001). At T2, participants in the CON group rated supplements as less efficient than those in the other groups (*Ps*≤.006, *ds*=0.64-1.04), which did not differ from each other.

Harmfulness increased significantly in the CON group from T0 to T2 (M_Δ_=19.82, *d*=0.84; *P*<.001) and to a smaller extent in the MIX group (M_Δ_=8.56, *d*=0.48; *P*=.004). At T2, these groups judged supplements as more harmful than both the PRO (*Ps*≤.007, *ds*=0.60 and 0.94) and control groups (*Ps*≤.01, *ds*=0.61 and 0.94).

Recommendability decreased significantly in the CON group from T0 to T2 (M_Δ_=−14.88, *d*=0.77; *P*<.001) and to a lesser degree in the MIX group (M_Δ_=−9.00, *d*=0.52; *P*=.008). At T2, the CON group reported lower recommendability than the PRO group (*P*<.001; *d*=0.82).

## Discussion

### Principal Findings

This study investigated how repeated exposure to polarized HRC influences explicit and implicit attitudes toward dietary supplements. Over 2 weeks of daily exposure to full-length articles, participants’ explicit attitudes shifted in the direction of the presented valence (H1b, H2b, H3b, partial H4b, H5b), while implicit attitudes remained stable. The expected moderation by initial attitudes (H7), mediation through engagement (ie, time spent reading, H8), and the hypothesized moderation by initial attitudes of the mediated effect (moderated mediation, H9) were not supported. Exploratory analyses of the 3 explicit attitude components (ie, perceived efficiency, harmfulness, and recommendability) showed distinct valence-dependent changes, indicating that repetition differentially shaped specific evaluative dimensions rather than producing uniform shifts.

Consistent with predictions derived from the illusory truth effect [13,16] and the mere exposure effect [12,14], repeated exposure to polarized HRC systematically modified explicit evaluations. Participants repeatedly exposed to favorable articles developed more positive explicit attitudes toward dietary supplements (H1b), while those exposed to negative articles developed more negative attitudes (H2b). The control group, who read unrelated content, showed no change, confirming that attitude shifts were due to informational valence rather than time or repeated testing. Repeated reading of congruent information, even when spread across distinct articles, appears sufficient to increase the perceived validity and desirability of the presented perspective.

These results align with prior findings that repetition enhances processing fluency, which is then misattributed to truth [26]. Because all articles were matched in length, structure, and evidential strength (no difference in trust ratings between groups, *F*_3, 224_=0.85; *P*=.47), the observed changes in explicit attitudes are unlikely to reflect persuasion via argument quality and instead point to fluency-driven propositional updating. The pattern observed in the mixed-exposure group provides further support for this interpretation. Participants in this condition consistently displayed negative shifts in their explicit attitudes (H3b), which, due to a technical ordering error, was the most recently encountered valence before each assessment point, suggesting that in contexts of informational ambivalence, recency effects can outweigh the cumulative balance of prior exposures. This is consistent with models of sequential belief updating showing that when conflicting information is presented sequentially and is comparable in evidentiary strength, recency exerts a disproportionate influence on judgments [43]. Nonetheless, because negative information is oriented toward more often and weighted more strongly than positive information (the “bad is stronger than good” phenomenon [44]), the present results cannot determine whether the observed pattern was driven primarily by recency, negativity dominance, or an interaction between the 2. Indeed, recent negative information may exert particularly strong influence because its temporal proximity and negative valence jointly increase its impact on judgment, making these mechanisms difficult to disentangle within the current design.

The exploratory analyses revealed that the CON group exhibited the strongest and most consistent shifts across all three evaluative dimensions: efficiency, harmfulness, and recommendability. The MIX group showed smaller but still significant increases in perceived harmfulness and decreases in recommendability, which suggests that repetition produces cumulative effects even when exposures are heterogeneous. These changes may reflect partial counterbalancing from the positive articles, the recency of the last negative exposure, or both. Because both harmfulness and recommendability displayed a consistent trend from T0 to T1 to T2 (with a significant change from T0 to T1 only for harmfulness, M_Δ_=–6.49; *P*=.04), the data suggest an additive negative exposure effect that was tempered, though not eliminated, by intermittent positive information. By contrast, efficiency increased modestly in the PRO group but was only slightly higher than in the control group at T2 (H4b, M_Δ_=8.76; *P*=.12). This suggests that positive claims about supplement benefits may be less impactful than warnings about potential harm. This asymmetry aligns with extensive evidence showing that negative information typically produces stronger and more persistent evaluative shifts than positive information [45,46]. Practically, these findings suggest that health-related judgments, especially those concerning perceived harm and social recommendation, are highly susceptible to repeated negative framing, whereas positive framing appears weaker and more fragile, but nevertheless offers some degree of protection when mixed in an environment saturated with negative exposure.

The observed effects extend prior findings based on brief or decontextualized statements [23,24], showing that fluency-driven evaluative shifts are not limited to aphoristic or slogan-like content. Instead, similar shifts arise from repeated exposure to full-length, naturalistic articles, suggesting that real-world news formats, despite their complexity and narrative structure, can still produce the same fundamental repetition-based distortions in explicit evaluations.

As online news consumption is driven primarily by negativity [47], HRC may be especially vulnerable to this dynamic due to its high personal relevance and the emotional weight carried by risk-related information. When algorithms amplify engagement signals, users are more likely to encounter repeated negatively framed health content, which our findings suggest can disproportionately influence evaluations of harmfulness and recommendability. In real-world settings where user interests and algorithmic curation jointly shape exposure, this may create a self-reinforcing loop of negative information. Practically, this underscores the value of deliberately incorporating balanced or positively framed health information. Further research on recency and timing is required to determine whether such content should be introduced strategically before important health decisions, in order to counteract the cumulative impact of negativity-heavy feeds and support more proportionate and evidence-aligned judgments.

In contrast to the clear shifts observed in explicit evaluations, implicit attitudes toward dietary supplements remained unchanged across the 2-week exposure period (H1-6a). This pattern aligns with contemporary evidence showing that changes in implicit evaluations depend strongly on the type, diagnosticity, and consistency of new information, the strength and age of prior attitudes, and the timescale of exposure [48]. Implicit attitudes are most likely to shift when the target is novel or weakly evaluated, when new information is strongly diagnostic or affectively potent, or when repeated pairings occur under tightly controlled conditions.

The present attitude object, dietary supplements, is familiar, frequently discussed, and carries a predominantly positive prior valence for our target population [49]. Implicit attitudes toward well-known targets tend to show reduced malleability and may be buffered by entrenched associative structures [50]. Additionally, because the articles were balanced in structure and intentionally nonsensational, the affective input may have been insufficient to trigger strong evaluative conditioning effects [51]. Repeated article exposure may therefore have lacked the associative shock or diagnostic weight needed to update established evaluations [33]

Another possibility is methodological: the IAT may be less sensitive to short-term changes when the target category is broad, multifaceted, or already strongly encoded [49]. Tasks such as the affect misattribution procedure (AMP [52]) or evaluative priming (EPT [53]) can detect subtler shifts in implicit evaluations of familiar objects and may therefore be better suited for future work on HRC.

Taken together, these findings suggest that while explicit evaluations of dietary supplements readily shift under repeated exposure to polarized information, implicit attitudes may require either more potent affective signals, more diagnostic or surprising information, or longer and more diverse exposure histories to exhibit measurable change. In real-world settings, such changes may arise only when repetition is accompanied by credible social endorsement, identity relevance, or experiential consequences. Dietary supplements, being familiar and frequently discussed in public discourse, may therefore represent an attitude domain in which everyday news exposure alone is unlikely to yield measurable changes in automatic evaluations.

Contrary to predictions, the hypothesized mediation by engagement, operationalized as time spent reading the assigned articles, was not supported (H8). Participants did not differentially invest time depending on the informational valence of the content. Similarly, the preregistered moderated mediation models testing whether baseline implicit or explicit attitudes moderated the relationship between group-specific exposure and engagement were nonsignificant (H9). This indicates that prior beliefs did not systematically shape how long participants engaged with the material. Our expectation that counterattitudinal information would be perceived as more diagnostic, thereby eliciting deeper engagement and stronger propositional updating, was therefore not supported (H7). The lack of mediation by engagement is consistent with findings that exposure alone, not depth of processing, is sufficient to shift explicit evaluations, because illusory truth effects operate primarily through familiarity and fluency rather than comprehension or elaboration [16,26]. However, our engagement measure was a coarse proxy, capturing only total reading time. More fine-grained measures such as eye-tracking, attention-based metrics (eg, attention allocation), or comprehension tests may better differentiate between superficial and elaborative processing in future work and offer clearer perspectives on their interaction with the illusory truth effect.

From a practical perspective, these findings suggest that stakeholders involved in health communication should consider patterns of repeated exposure in addition to the accuracy of individual messages. Public health agencies, healthcare professionals, and science communicators may benefit from disseminating evidence-based information repeatedly over periods of days or weeks, rather than relying on single-exposure campaigns. Examples include scheduled reposting of evidence-based content, recurring newsletter messages, and push notifications that direct users to trustworthy health information sources. Because repeated exposure influenced explicit evaluations even when participants read relatively lengthy articles, interventions aimed at improving public understanding of health topics may benefit from emphasizing sustained visibility rather than one-time corrections.

At the same time, platform designers and policymakers should recognize that recommendation systems may influence health attitudes through cumulative exposure patterns. In addition to content moderation and fact-checking, platforms could monitor whether users are repeatedly exposed to predominantly favorable or unfavorable health content and test strategies that increase informational diversity. For example, recommendation systems could periodically introduce evidence-based content presenting alternative perspectives, while A/B testing could be used to compare the effects of different exposure frequencies and content distributions on user attitudes and engagement. Such approaches may help reduce the formation of health-related echo chambers while preserving access to accurate information.

### Limitations and Further Directions

Although several methodological limitations and directions for further research have already been noted in interpreting the findings, a few broader limitations and directions warrant explicit consideration.

This study sought to improve ecological validity in the investigation of repeated exposure by using full-length, naturalistic articles rather than brief statements or isolated claims. Although this approach provides a closer approximation to real-world health communication, the exposure environment still differed from genuine online news ecosystems. Participants read a single text-only article per day, whereas everyday exposure is rapid, interleaved, multimodal, and often shaped by social cues such as likes, comments, or peer sharing. Future research should incorporate more dynamic presentations, such as simulated algorithmic feeds, social endorsement signals, or mixed-content timelines, in order to capture the attentional competition and contextual layering characteristic of actual digital media environments.

A second limitation concerns the MIX condition. Although it was designed to provide balanced exposure, a technical error produced a fixed sequence in which negative content always immediately preceded each assessment. This systematic recency constraint limits our ability to interpret whether attitude trajectories reflected cumulative exposure, recency-driven updating, or an interaction of the two. Fully counterbalanced designs and stimuli that integrate both positive and negative information within the same article would allow future studies to disentangle additive effects from recency and negativity biases more precisely. A particularly informative design would orthogonally manipulate overall exposure balance and final exposure valence, for example by comparing sequences with the same number of positive and negative articles but ending either with a positive or a negative article. This would clarify whether mixed exposure effects are driven by cumulative balance, the valence of the most recent exposure, or whether negative final exposures exert disproportionate influence compared with positive final exposures.

A third limitation is that the sample consisted primarily of university students, which restricts generalizability. Young adults differ from older or more medically vulnerable populations in terms of prior familiarity with dietary supplements [49], susceptibility to misinformation [54], and online consumption patterns. Future work should recruit more diverse samples, including older adults, individuals with chronic health conditions, and frequent consumers of HRC on social media platforms.

Another limitation is that this study focused exclusively on attitudinal outcomes and did not assess behavioral consequences. Although repeated exposure influenced explicit evaluations of dietary supplements, it remains unclear whether these changes would translate into actual health-related behaviors. This question is particularly important given the absence of changes in implicit attitudes, which are often associated with more automatic forms of behavior. Future studies should incorporate behavioral measures, such as purchasing intentions, sharing intentions (eg, willingness to share supplement-related articles with friends or social media contacts), supplement consumption, or behavioral preference tasks. For example, participants could choose between a chance to win dietary supplements or an alternative reward of comparable value (eg, cereal bars), or between receiving a guide to high-quality supplement producers and a guide to healthy cooking. In addition, follow-up assessments conducted 1 and 3 months after the intervention could examine whether exposure predicts subsequent information-seeking behavior, supplement use, purchasing decisions, or the sharing of supplement-related content. Such measures would help determine whether repeated exposure to polarized HRC translates into meaningful behavioral outcomes.

A final limitation concerns the duration of the exposure period. Although the 2-week design allowed us to examine attitude trajectories at multiple assessment points while maintaining participant engagement with a demanding daily exposure protocol, it remains unclear whether the observed changes in explicit attitudes would persist, accumulate, or diminish over longer periods of exposure. Future studies should examine longer exposure schedules and include follow-up assessments to determine the durability of these effects and their relevance to real-world information environments.

### Conclusions

The present findings show that repeated exposure to polarized HRC can shift explicit but not implicit attitudes, even when the content consists of long, naturalistic articles rather than isolated statements.

The explicit-implicit dissociation observed here offers a nuanced perspective on the potential risks of repeated exposure. On the one hand, the absence of changes in implicit attitudes suggests that underlying automatic associations remained stable, which may act as a buffer against drastic behavioral shifts. On the other hand, explicit attitudes guide reflective choices, risk assessments, and help-seeking behaviors, meaning that incremental changes in perceived harmfulness or recommendability could still influence consumer decisions, conversations with peers, or trust in official guidance.

In algorithmic news environments, where repetition is driven not by editorial intention but by engagement metrics, many such subtle explicit shifts may accumulate over time from various sources. This suggests that efforts to promote evidence-aligned health judgments should consider not only the accuracy of content but also the informational environments that shape familiarity and fluency. Supporting healthier digital ecosystems may require designing feeds that balance valence, introduce corrective or contextualizing information, and avoid reinforcing negativity-driven loops. In doing so, we may help preserve reflective health judgments without assuming that deeper evaluative change necessarily follows from repetition alone.

From an infodemiological perspective, the findings suggest that repeated exposure itself may represent an important mechanism through which online health information environments shape user evaluations. Consequently, exposure diversity and timing may deserve consideration alongside content accuracy in efforts to mitigate misinformation risks.

## Supplementary material

10.2196/88632 Multimedia Appendix 1 Methodological information, descriptive statistics, and additional analyses.

10.2196/88632 Multimedia Appendix 2 Full text of the article stimuli used in the repeated exposure intervention.

## Abbreviations

AMP: affect misattribution procedure
ANCOVA: analysis of covariance
BootCI: bias-corrected bootstrapped confidence interval
CON: negative exposure condition/group
EPT: evaluative priming task
HRC: health-related content
IAT: Implicit Association Test
MIX: mixed exposure condition/group
PRO: positive exposure condition/group
VAS: visual analog scale
