# Supplementary material for: Impact of Repeated Exposure to Polarized Health-Related News on Explicit and Implicit Attitudes Toward Dietary Supplements: Online Experimental Study
Source: JMIR Infodemiology. 2026 Jul 27;6:e88632. doi: 10.2196/88632 (PMC13404937; doi:10.2196/88632)
Supplement: Multimedia Appendix 1 [file infodemiology-v6-e88632-s001.docx]

Multimedia Appendix 1

P = Positive article (PRO)

C = Negative article (CON)

S = Space article (Control)

* = Article included in the MIX group

# P1*

**Can you trust dietary supplements?**

The majority of adults in the United States take one or more dietary supplements either every day or occasionally. Today’s dietary supplements include vitamins, minerals, herbals and botanicals, amino acids, enzymes, and many other products. Dietary supplements come in a variety of forms: traditional tablets, capsules, and powders, as well as drinks and energy bars. Popular supplements include vitamins D and E; minerals like calcium and iron; herbs such as echinacea and garlic; and specialty products like glucosamine, probiotics, and fish oils.

If you don’t eat a nutritious variety of foods, some supplements might help you get adequate amounts of essential nutrients

Scientific evidence shows that some dietary supplements are beneficial for overall health and for managing some health conditions. For example, calcium and vitamin D are important for keeping bones strong and reducing bone loss; folic acid decreases the risk of certain birth defects; and omega-3 fatty acids from fish oils might help some people with heart disease.

Many supplements contain active ingredients that can have strong effects in the body.

Many supplements contain active ingredients that can have strong effects in the body. Always be alert to the possibility of unexpected side effects, especially when taking a new product.

Dietary supplements are complex products. The FDA has established good manufacturing practices (GMPs) for dietary supplements to help ensure their identity, purity, strength, and composition. These GMPs are designed to prevent the inclusion of the wrong ingredient, the addition of too much or too little of an ingredient, the possibility of contamination, and the improper packaging and labeling of a product. The FDA periodically inspects facilities that manufacture dietary supplements.

In addition, several independent organizations offer quality testing and allow products that pass these tests to display their seals of approval. These seals of approval provide assurance that the product was properly manufactured, contains the ingredients listed on the label, and does not contain harmful levels of contaminants. These seals of approval do not guarantee that a product is safe or effective. Organizations that offer this quality testing include:

U.S. Pharmacopeia

ConsumerLab.com

NSF International

Once a dietary supplement is on the market, the FDA monitors information on the product’s label and package insert to make sure that information about the supplement’s content is accurate and that any claims made for the product are truthful and not misleading. The Federal Trade Commission, which polices product advertising, also requires all information about a dietary supplement product to be truthful and not misleading.

The federal government can take legal action against companies and Web sites that sell dietary supplements when the companies make false or deceptive statements about their products, if they promote them as treatments or cures for diseases, or if their products are unsafe.

# C1*

**Can you trust dietary supplements?**

If you don’t eat a nutritious variety of foods, some supplements might help you get adequate amounts of essential nutrients. However, supplements can’t take the place of the variety of foods that are important to a healthy diet.

Supplements need more study to determine their value. The U.S. Food and Drug Administration (FDA) does not determine whether dietary supplements are effective before they are marketed.

Supplements are most likely to cause side effects or harm when people take them instead of prescribed medicines or when people take many supplements in combination. Some supplements can increase the risk of bleeding or, if a person takes them before or after surgery, they can affect the person’s response to anesthesia. Dietary supplements can also interact with certain prescription drugs in ways that might cause problems. Here are just a few examples:

Vitamin K can reduce the ability of the blood thinner Coumadin® to prevent blood from clotting.

St. John’s wort can speed the breakdown of many drugs (including antidepressants and birth control pills) and thereby reduce these drugs’ effectiveness.

Antioxidant supplements, like vitamins C and E, might reduce the effectiveness of some types of cancer chemotherapy.

Keep in mind that some ingredients found in dietary supplements are added to a growing number of foods, including breakfast cereals and beverages. As a result, you may be getting more of these ingredients than you think, and more might not be better. Taking more than you need is always more expensive and can also raise your risk of experiencing side effects. For example, getting too much vitamin A can cause headaches and liver damage, reduce bone strength, and cause birth defects. Excess iron causes nausea and vomiting and may damage the liver and other organs.

Be cautious about taking dietary supplements if you are pregnant or nursing. Also, be careful about giving them (beyond a basic multivitamin/mineral product) to a child. Most dietary supplements have not been well tested for safety in pregnant women, nursing mothers, or children.

The term “natural” doesn’t always mean safe. A supplement’s safety depends on many things, such as its chemical makeup, how it works in the body, how it is prepared, and the dose used. Certain herbs (for example, comfrey and kava) can harm the liver.

Dietary supplements are products intended to supplement the diet. They are not drugs and, therefore, are not intended to treat, diagnose, mitigate, prevent, or cure diseases.

In general, the FDA regulations for dietary supplements are different from those for prescription or over-the-counter drugs. Unlike drugs, which must be approved by the FDA before they can be marketed, dietary supplements do not require premarket review or approval by the FDA. While the supplement company is responsible for having evidence that their products are safe and the label claims are truthful and not misleading, they do not have to provide that evidence to the FDA before the product is marketed.

# P2*

**The utility of dietary supplements**

Some people don’t get enough vitamins and minerals from their daily diet, and their doctors may recommend a supplement. Dietary supplements may provide nutrients that might be missing from your daily diet.

People over age 50 may need more of some vitamins and minerals than younger adults do

Calcium. Calcium works with vitamin D to keep bones strong at all ages. Bone loss can lead to  in both older women and men. Calcium is found in milk and milk products (fat-free or low-fat is best), canned fish with soft bones, dark-green leafy  like kale, and foods with calcium added, like breakfast cereals.

Vitamin D. Most people’s bodies make enough vitamin D if they are in the sun for 15 to 30 minutes at least twice a week. But, if you are older, you may not be able to get enough vitamin D that way. Try adding vitamin D-fortified milk and milk products, vitamin D-fortified cereals, and fatty fish to your diet, and/or use a vitamin D supplement.

Vitamin B6. This vitamin is needed to form red blood cells. It is found in potatoes, bananas, chicken breasts, and fortified cereals.

Vitamin B12.Vitamin B12 helps keep your red blood cells and nerves healthy. While older adults need just as much vitamin B12 as other adults, some have trouble absorbing the vitamin naturally found in food. If you have this problem, your doctor may recommend that you eat foods like fortified cereals that have this vitamin added, or use a B12 supplement.

Here are some common sources of antioxidants that you should be sure to include in your diet:

Beta-carotene- and vegetables that are either dark green or dark orange

Selenium-Seafood, liver, meat, and grains

Vitamin C-Citrus fruits, peppers, tomatoes, and berries

Vitamin E-Wheat germ, nuts, sesame seeds, and canola, olive, and peanut oils

What do we know about the effectiveness of dietary supplements?

Studies have found that some dietary supplements may have some benefit, such as melatonin for jet lag, and others may have little or no benefit, such as ginkgo for dementia.

Taking a multivitamin is unlikely to pose any health risks.

Although some herbal and mineral compounds have been used for hundreds of years to treat health conditions, today dietary supplement manufacturers are not legally allowed to say their products cure, treat or prevent disease.  Supplement makers can say their products support health or contribute to well-being.

Some supplements can play an important role in health. For example, calcium and vitamin D are important for keeping bones strong. Pregnant women can take the vitamin folic acid to prevent certain birth defects in their babies.

# C2*

**Do you need a dietary supplement?**

Dietary supplements are substances that you might use to add nutrients to your diet or reduce your risk of health problems, such as osteoporosis or arthritis. Dietary supplements come in pill, capsule, powder, gel, extract, or liquid form. They may contain vitamins, minerals, fiber, amino acids, herbs, or other plants or enzymes. Sometimes, the ingredients in dietary supplements are added to foods, including beverages. You don’t need a doctor’s prescription to buy dietary supplements.

Eating a variety of healthy foods is the best way to get the nutrients you need.

Wondering if you need a dietary supplement? You probably do, but the answer is usually no. Ask yourself why you think you might want to take a dietary supplement. Are you worried about not getting enough nutrients? Has a friend, neighbor, or someone in an advertisement suggested you take one? Some ads for dietary supplements in magazines, online, and on television seem to promise that these supplements will make you feel better, prevent you from getting sick, or even help you live longer. Often, these claims are based on little quality scientific research, and in some cases, they are completely unfounded. Supplements can be expensive, potentially harmful, or simply not helpful.

Current research suggests that high doses of antioxidant supplements will not prevent chronic diseases such as heart disease or diabetes. In fact, some studies have shown that taking high doses of antioxidants may be harmful.

Researchers are looking into the use of herbal supplements to prevent or treat some health problems. It is too early to know whether herbal supplements are safe and effective. Some studies have shown no benefit or have shown benefits similar to a placebo effect.

What do we know about the effectiveness of dietary supplements?

 Studies generally show little or no benefit. One example is the much-vaunted effect of ginkgo for treating and preventing dementia, which has been shown to be null. Ask your doctor for evidence before buying.

 The supplements you buy in stores or online may differ in important ways from the products tested in studies.

 Most research shows that taking multivitamins does not lead to a longer life, slow cognitive decline, or reduce the chances of cancer, heart disease, or diabetes.

Some products marketed as dietary supplements—mostly promoted for weight loss, sexual performance, and bodybuilding—may contain unapproved drugs or other ingredients not listed on the label. Some of these ingredients may be unsafe.

People buy vitamins and other nutritional supplements with the goal of improving their health, but they don’t know exactly what to look for or what’s inside the bottle. Just because a supplement is labeled “all-natural” doesn’t mean it’s safe or effective. Caution is advised.

# P3*

**A ranking of the five most popular supplements taken to aid sleep, based on sales volume and popularity.**

**Ranking of the most popular dietary supplements taken to aid sleep**

A ranking of the five most popular supplements taken to aid sleep, based on sales volume and popularity.

1. MELATIONIN

Melatonin is a hormone your body produces naturally, which signals your brain that it’s time to sleep

This hormone’s cycle of production and release is influenced by time of day - melatonin levels naturally rise in the evening and fall in the morning.

For this reason, melatonin supplements have become a popular sleeping aid, particularly in instances where the melatonin cycle is disrupted, such as jet lag

Melatonin may improve overall sleep quality in individuals suffering from sleep disorders. Specifically, melatonin appears to reduce the time people need to fall asleep and increase the total amount of sleep time

While there are also studies that did not observe melatonin had a positive effect on sleep, they were generally few. Those that did observe beneficial effects generally provided participants 3–10 mg of melatonin before bedtime.

2. Valerian Root
Most experts recommended this herb to reduce the amount of time it takes to nod off. According to the NIH, no single compound in valerian has been identified as the active agent. However, the NIH reports that valerian seems to have sedative properties, and it may increase the amount of GABA (gamma-aminobutyric acid), a compound in the brain that prevents the transmission of nerve impulses. Valerian seems to be especially effective when combined with hops, according to a 2007 study.

3. 5-HTP

A compound derived from the amino acid L-tryptophan, the supplement also is used to enhance mood and decrease appetite. Laurie Steelsmith, a licensed naturopathic physician, does not recommended 5-HTP for those on antidepressant medications. Steelsmith says that 5-HTP acts as a precursor to serotonin, which is a neurotransmitter that is essential for a good night’s sleep. “It is better than L-tryptophan because 5-HTP can cross the blood-brain barrier and thus increase serotonin in the brain.” A small 2009 study of 18 people found that those who took a product combining 5-HTP and GABA needed less time to fall asleep, slept longer and reported improved sleep quality.

4. Magnesium
Along with contributing to a good night’s sleep, this light, silvery metallic element is an oft-overlooked nutrient that helps maintain normal muscle and nerve function, keeps heart rhythm steady, supports a healthy immune system, and keeps bones strong. Magnesium also helps regulate blood sugar levels, and promotes normal blood pressure, according to the NIH. Lack of magnesium inhibits nerve cell communication, which leads to cell excitability. The result: a stressed and nervous person. Several older studies show that magnesium can improve sleep quality and reduce nocturnal awakenings.

5. Sleep Supplement Formula: Natural Health Natural Sleep Plus Calcium
is formulated with 3 parts magnesium to 2 parts calcium, which will prevent a surplus of calcium robbing the body's vital magnesium supply and avoid the symptoms associated with depleted levels of magnesium. This superior formulation includes potassium, boron, and vitamin D for better absorption of these key minerals, building strong bones, regulating hormones, and more. You can't create a better calcium-magnesium formula.

# C3*

**Debunking Sleep Myths: Are Natural Sleep Aids Safe?**

A sleepless night can leave you cranky, lethargic, and feeling out of sorts in the morning. When those wide-awake nights starts happening more and more frequently, the idea of taking a natural sleep aid may seem like a reasonable way to cope. But while these aids are often promoted as safe, effective ways to treat , they may not be the best choice. Talking to your doctor before taking any natural sleep aid is a must for these four reasons.

1. Natural sleep aids are unregulated.

The Food and Drug Administration (FDA) does not regulate dietary supplements (which include natural sleep aids like valerian and ) for safety, quality, effectiveness, or truth in marketing claims. In fact, in 71 percent of melatonin supplements on the market, the melatonin content doesn’t come within 10 percent of its labeled claim.  It’s best to talk with your doctor about the options and how they might affect you.

2. They’re not ok for everyone.

It’s a misconception that because sleep aids are labeled as natural, they’re safe for anyone to take. Certain groups of people need to be cautious. For example, melatonin may have an effect on an adolescent’s developing reproductive, cardiovascular, immune, and metabolic systems.  Women who are pregnant or trying to become pregnant are advised to avoid melatonin because there have been no long-term studies on its effects on mothers and developing fetuses.

A natural sleep aid could also interact negatively with medication you’re currently taking. For instance, in laboratory studies melatonin supplements reduced the effectiveness of antidepressants desipramine and fluoxetine.  Melatonin has also been shown to decrease the effectiveness of certain blood pressure medications.  Talk with your doctor before taking a natural sleep aid to ensure that your medications won’t be compromised.

3. Natural sleep aids won’t cure insomnia.

Sometimes, sleeplessness is a short-term problem with an identifiable cause, such as jet lag or a physical injury that’s keeping you awake. In those cases, your doctor may feel a natural sleep aid is worth a try. But for those suffering from long-term sleep issues, it’s important to get to the root of the situation. Your doctor may ask you questions about your sleep habits, how many hours you sleep on weeknights and weekends, and what your bedroom environment is like. With this information, a physician can make specific recommendations for improving the quality of your sleep in the long term, rather than relying on short-term relief from natural sleep aids.

4. The lasting effects are unknown.

While a few studies have shown melatonin to be a moderately effective sleep aid, research on the long-term effects of natural sleep aids is sparse.  And since the information is lacking, talking with a medical expert is the best way to ensure your safety. Your doctor can help determine how a natural sleep aid may or may not benefit you, and whether given your lifestyle and sleep issues, trying one makes sense.

# P4

**Are you looking for the best sleep supplements that are safe and scientifically tested to help you sleep better?**

Chances are if you are an adult, you’ve had trouble sleeping. Recent studies have found that 1 out of every 3 adults in the U.S. do not get the recommended seven hours of sleep regularly, while millions of adults around the globe suffer from sleeplessness and  nightly. The good news is, there are proven  for deep sleep that really work.

Whether you want to fall asleep faster, beat jet lag, adjust to shift work, or just have trouble sleeping, taking a natural sleep supplement before bed can definitely help you get a better night’s sleep.

Lemon balm

Lemon Balm, also known as ‘Melissa officinalis’, has been used to treat anxiety and sleep in restless children. In a 2006 study involving over 900 children suffering from dyssomnia and lemon balm’s therapeutic ability, over 80% of the child participants experienced an improvement.

Taking lemon balm – dosage for sleep: A 2011 study that found that 300 mg of standardized Lemon balm twice a day can be effective at reducing stress and improving sleep in adults suffering mild to moderate sleep disorders.

Vitamin B-6

 helps to regulate sleep patterns. By failing to obtain an adequate amount of vitamin B-6 in your diet, you may limit the amount of serotonin in your body, potentially leading to disturbed sleep patterns and insomnia. A deficiency in B6 may cause insomnia and difficulty sleeping. B6 has proven to stimulate the brain during sleep phases of REM, and individuals often say it gives them more vivid dreams.

Taking B-6 – dosage for sleep: The recommended intake of B-6 each day is 1-2mg a day. Your requirement increases as you get older. For vitamin B6 deficiency that may be causing sleeplessness: in most adults, the typical dose is 2.5-25 mg daily for three weeks then 1.5-2.5 mg per day thereafter. In women taking birth control pills, the dose is 25-30 mg per day, according to The Natural Medicines Comprehensive Database

 GABA

GABA (gamma butyric acid) is a naturally occurring chemical compound produced in the  that helps to calm the excitability of neurons. In a 2013 study on elderly men with frequent night urination, GABA proved to enhance sleep length, quality and greatly curbing nighttime urination. And it cuts your wait time for Z’s in half. in a study published back in 2017 that took place at UCLA School of Medicine, people who were given a GABA supplement were able to fall asleep in almost less than half the time it took those taking placebos and increased their time spent sleeping by approximately 73%.

Taking GABA – dosage for sleep: A 100mg daily dosage of GABA was found to help people fall asleep more quickly, according to a study conducted in 2015.

# C4

**Follow these warnings if you use supplements to help you sleep**

Many types of supplements claim to help you sleep. For example: Valerian root. The root of this tall, flowering plant is said to help people fall asleep and relieve anxiety and stress. It has been used as a medicinal herb since ancient Rome.

Chamomile. This daisy-like flower has also been used for thousands of years. It is taken in both pill and tea form (many people drink a cup of chamomile tea at bedtime). It is considered mild and safe to help you fall asleep. But some people have an allergic reaction to chamomile (especially those who are allergic to ragweed).

Melatonin. This supplement comes from a lab, not a plant. It is a synthetic version of the human hormone that helps regulate your sleep-wake cycle. But Dr. Epstein says it’s not a sleep aid. “It makes people a little sleepy, but it has a much greater effect on shifting the timing of sleep phases,” he explains. Even for adults, the unregulated nature of the supplement offers reason to reconsider. While synthetic melatonin is “chemically identical” to natural melatonin, commercially available supplements “often contain fillers, inerts, and other ingredients that can cause effects that you wouldn’t expect with natural melatonin,” says Clete Kushida, a sleep researcher at Stanford University School of Medicine. . Dr. Epstein recommends taking one to three milligrams of melatonin two to three hours before bed if you’re trying to better align your sleep cycle due to jet lag or a night shift job. He says melatonin is safe to take long-term.

Warning

Although supplements are widely taken to help people sleep, we don’t really know if they work.

“There is essentially no data on any herbal supplement that shows efficacy, except for a minor beneficial effect of valerian root,” says Dr. Epstein.

Many supplements can have minor side effects, such as headaches, dizziness, or nausea. Or they can increase the effects of alcohol or other medications you’re taking, such as other sleep medications. Perhaps the biggest concern is that the FDA doesn’t regulate supplements, so there’s no way to know if a pill contains what its manufacturer claims.

What to do?

If you want to take an over-the-counter sleep aid or dietary supplement, talk to your doctor or pharmacist to make sure it won’t interact with any medications you’re taking.

If you're having more than the occasional sleepless night, it may be time to figure out what's causing your problems. "Most sleep problems can be corrected without medication," says Dr. Epstein. "But it may take multiple approaches. Sleep problems are often caused by many things, not just one thing that can be fixed with a pill."

# P5

**Best Supplements and Vitamins for Weight Loss**

You’ve tried the grapefruit juice diet, the cabbage soup diet, the baby food diet, cleanses, and detoxes, yet you’re still not seeing the results you want. It’s not surprising, as there’s a lot of debate on the effectiveness of . So what’s left to try? Vitamins.

“As part of a healthy living plan that includes clean eating, exercise, and stress management, we find that vitamins and minerals can play a role in weight loss and weight management,” say , authors of the book . “Many, if not most of us, have nutrient needs that are unmet by diet alone. When we replenish these deficiencies with the right vitamins and minerals, in proper doses, then we can satisfy our body’s nutrient cravings and in turn, reduce our unhealthy eating.”

Many of the doctors’ patients report that once they begin a regimen that includes the right vitamins for their individual needs, they’re able to eat less while making better food choices, and find they have more energy to exercise and plan meals. In addition, they tend to sleep better-and that also helps with weight control. Sold? Here are the top vitamins for weight loss.

Vitamin D

When you are low in vitamin D, your body will convert sugar to fat instead of energy, warns  a doctor of naturopathy, clinical nutritionist, and chiropractic neurologist. Vitamin D levels are lower in overweight individuals as well as in those who are physically inactive and this deficiency is a common cause of . A 2018 study published in the International Journal of Preventive Medicine found that  was accompanied with higher risk of type 2 diabetes, cardiovascular diseases, hypertension, and obesity. You may think you’re getting your daily dosage of vitamin D from the sun, but also make sure that you’re  too.

Iron

We’re facing two major health threats worldwide: Iron deficiency and obesity. And according to a 2014 study in Nutrients,  are connected-with weight loss a key factor in combating the deficiency. Women are at particular risk, as they lose iron throughout their lives due to menstruation. “Those who are iron deficient may have trouble losing weight, and therefore, appropriately replenishing iron levels can help with weight loss,” according to Dr. Levitan and Dr. Block. “Typically iron should be paired with vitamin C to aid in absorption. In addition, iron plays a role in energy levels-when we feel energized, we are more inclined to exercise, which, of course, can assist with any weight loss efforts.”

# C5

**Supplements and vitamins for weight loss?**

What are weight loss supplements and what do they do?

The proven ways to lose weight are to eat healthy foods, cut calories, and exercise. But making these lifestyle changes isn’t easy, so you may wonder if taking a dietary supplement that’s marketed for weight loss could help.

The sellers of these supplements may claim that their products help you lose weight by blocking the absorption of fat or carbohydrates, reducing your appetite, or speeding up your metabolism. But there’s little scientific evidence that weight loss supplements work. Many are expensive, some may interact or interfere with medications, and a few may be harmful.

If you’re considering taking a dietary supplement to lose weight, talk to your doctor. This is especially important if you have high blood pressure, diabetes, heart disease, liver disease, or other medical conditions.

Weight loss supplements contain many ingredients—such as herbs, fiber, and minerals—in varying amounts and combinations. Sold in capsules, tablets, liquids, and powders, some products have dozens of ingredients.

It can be tricky to figure out if these ingredients are really helping you lose weight safely. Most products contain more than one ingredient, and ingredients can work differently when combined. You might be surprised to learn that weight loss supplement manufacturers rarely conduct human studies to find out if their product works and is safe. And when studies are done, they usually involve only a small number of people taking the supplement for just a few weeks or months. To know whether a weight loss supplement can help people safely lose weight and keep it off, larger groups of people need to be studied over a much longer period of time.

Can weight loss supplements be harmful?

Weight-loss supplements, like all dietary supplements, can have harmful side effects and can interact with prescription and over-the-counter medications. Many weight-loss supplements contain ingredients that have not been tested in combination with each other, and their combined effects are unknown. Tell your healthcare providers about any weight-loss supplements or other supplements you are taking. This information will help them work with you to prevent supplement-drug interactions, harmful side effects, and other risks.

Fraudulent and Adulterated Products

Be very wary of weight-loss supplements with tempting claims such as “magic weight-loss pill,” “melt fat,” and “lose weight without diet or exercise.” If the claim sounds too good to be true, it probably is. These products may not help you lose weight and could be dangerous.

# P6

**What can help in the fight against extra kilos?**

Some of us are predisposed to excess weight due to heredity or other factors. Even with a healthy diet and regular exercise some of us still struggle to keep the weight off. If you’re one of these people then you know how frustrating the weight merry-go-round can be.

There are certain vitamins, minerals, and nutrient concentrations found in nature that, when taken along with a  and , have the ability to rev your metabolism and help your body process macronutrients more efficiently. If you’re looking to lose fat, stock up on these supplements.

Green tea extract

“Green tea extract may increase fat oxidation and even stimulate brown adipose tissue thermogenesis-special fat cells that can actually help speed up your metabolism, which is good news for anyone looking to lose body fat,” says Jalali. The key is in the plant’s phytochemicals, particularly epigallocatechin gallate (ECGC), which  found, at low doses, can increase your  by 33 percent. Jalali advises looking for a supplement with 50% EGCG and 90% polyphenols for the best burn.

Glutamine

The most abundant amino acid naturally produced by your body, glutamine is found in the largest amounts in your muscles, helping repair them, maintain , and stabilize your blood sugar.  found that without changing their diets at all, people lost weight after taking glutamine supplements for four weeks, likely because it helped boost glucose metabolism.

Chitosan

This fiber, commonly called “shark cartilage,” comes from the exoskeletons of insects and crustaceans. Research is mixed, but  found that when people took chitosan twice a day with food, they had significantly lower BMIs and body fat as well as less bloating and  than people who took a placebo. It doesn’t have magical fat burning abilities, but, because it’s a type of dietary fiber, it may help keep you full longer, which in turn leads to you eating less, Cohen explains.

Cayenne pepper

The research in support of cayenne pepper for weight loss is on fire: Taking half a teaspoon in a capsule can help you eat fewer calories later in the day and crave sweet and salty snacks less, according to . Meanwhile,  have found that eating meals with capsaicin-the root of pepper’s heat-helps increase your metabolism by 15 to 20% for up to two hours after. Plus,  earlier this year found that capsinoids can help activate white and brown fat cells, helping to better regulate your metabolism. The secret is in the fact that capsaicin activates your sympathetic nervous system, which is what controls your adrenaline and fight or flight response, explains Jalali (also why you feel warmer when you eat the heat).

# C6

**The Problems of Using Supplements to Lose Weight**

How Do Weight Loss Supplements Work?

If you're looking for dietary supplements to help you lose weight, you'll have no shortage of options. Companies that market these products claim that the ingredients in their supplements can help you achieve results like these:

• Suppress your appetite (bean, bee pollen, fennel) • Make you feel full before you overeat (guar gum, psyllium) • Boost your metabolism (caffeine, guarana, synephrine, B vitamins) • Slow down the body's production of fat (green tea, hydroxycitric acid, flaxseed) • Help your body absorb fat from the foods you eat (chondroitin)

Dietary supplements come in almost any form you can take orally - from pills and capsules to powders, liquids, and teas. Some products are taken with meals, while others are taken in place of meals. Over-the-counter diet pills are supposed to help you lose weight by boosting your metabolism, your body’s energy-using system. The main ingredient in metabolic support products like Dexatrim is caffeine, a central nervous system stimulant that can help you burn fat through a process called thermogenesis. But the risks of taking these stimulants may outweigh the benefits.

Why are weight-loss treatments dangerous? Many dietary supplements are harmless, and some can even be effective at making you feel full, burning fat, or boosting your metabolism. But some of the popular ingredients in weight loss products have been banned by the FDA due to harmful side effects such as:

• Increased heart rate

• High blood pressure

• Agitation

• Diarrhea

• Insomnia

• Kidney problems

• Liver damage

• Rectal bleeding

Ephedra – Banned

Once widely sold as an ingredient in dietary supplements, the Chinese herbal stimulant ephedra was banned in 2004 due to evidence that its use could increase the risk of heart attack or stroke. In 2005, a lower court ruled that ephedra could be used in small doses. In 2006, a federal appeals court reinstated the FDA’s original ban, ruling that ephedra was too dangerous to be used as a supplement in any dosage.

Hydroxycut – Recalled and Banned Some weight-loss products can cause severe damage to the kidneys, liver, and other vital organs. Hydroxycut products were banned and recalled in 2009 due to reports of serious side effects, including hepatitis and jaundice. One person who took these fat-burning supplements died; another required a liver transplant.

Taking too much of a dietary supplement or combining supplements can be extremely dangerous. An overdose of stimulant products can raise your blood pressure to dangerously high levels, putting you at risk for a heart attack or stroke. Taking fat-blocking supplements with laxatives or diuretics can cause diarrhea, fluid loss, and an electrolyte imbalance. Overusing products that pose a risk of liver or kidney damage only increases the possibility of life-threatening organ failure.

# P7*

**The Science of Immunity: How 5 Supplements Help You Fight Influenza**

When flu season approaches, strengthening the body's natural defenses becomes a priority. While annual vaccines and healthy lifestyle habits remain the foundation of defense, clinical research shows that specific natural supplements can optimize your immune response against the Influenza virus.

Here is what peer-reviewed science says about how these five natural allies support the body in preventing and recovering from Influenza.

1. Garlic: Activating Primary Viral Defenses

Garlic contains specialized organosulfur compounds, most notably allicin, which is released when the clove is crushed. Allicin is highly valued in immunology for its direct antiviral and immune-stimulating properties.

Clinical trials show that daily garlic supplementation actively enhances the function of natural killer (NK) cells and T-cells—the body's primary defense forces against viral invaders. By boosting these immune cells, garlic helps the body rapidly detect and neutralize the Influenza virus before it can aggressively replicate throughout the respiratory tract.

2. Ginger: Blocking Viral Attachment

Used for centuries to treat respiratory illness, ginger contains highly active compounds known as gingerols and shogaols.

Virology studies demonstrate that fresh ginger extract has explicit anti-influenza properties. Specifically, it can prevent the Influenza virus from attaching to and entering the mucosal cells of the human airway (*Journal of Ethnopharmacology*). Additionally, ginger’s natural anti-inflammatory traits help soothe the intense throat inflammation and chest soreness characteristic of a flu infection.

3. Turmeric: Balancing the Inflammatory Storm

Turmeric owes its therapeutic power to curcumin, a polyphenol extensively researched for its ability to regulate the body’s immune pathways.

A major complication of severe Influenza is an overreactive immune response that causes systemic aches, chills, and fatigue. Clinical data shows that curcumin acts as a molecular regulator, downregulating the inflammatory pathway known as *NF-κB*. By stabilizing this response, curcumin helps the body fight the virus efficiently while mitigating the harsh, draining physical symptoms of the flu.

4. Selenium: Supporting Vaccine Efficacy and Cellular Repair

Selenium is an essential trace mineral required to synthesize selenoproteins, which form the backbone of the body’s internal antioxidant defense system.

Influenza viruses cause severe oxidative stress, damaging healthy lung tissue. Research shows that maintaining optimal selenium levels keeps these protective selenoproteins fully active, shielding respiratory cells from viral damage. Furthermore, nutritional data suggests that adequate selenium levels support robust antibody production following flu vaccinations, maximizing long-term protection.

5. Ground Flaxseed: Easing Respiratory Distress

Ground flaxseed is a premier plant-based source of alpha-linolenic acid (ALA), an essential omega-3 fatty acid that supports cell membrane integrity.

During an active influenza infection, the bronchial pathways experience severe inflammatory stress, resulting in deep coughing. Clinical research highlights that the omega-3 fatty acids found in flaxseed help modulate systemic inflammation, effectively reducing airway hypersensitivity and easing respiratory strain.

The Bottom Line

Incorporating scientifically backed supplements like garlic, ginger, turmeric, selenium, and ground flaxseed into your wellness routine can safely prime your immune system, minimize influenza-related inflammation, and help your body recover its strength faster.

# C7

**Influenza and Supplements: What You Need to Know**

Why do people with influenza use supplements?

Many people with the flu use supplements to help fight the virus or to make them feel better. Most people use supplements along with conventional treatments, such as rest and antiviral medications. But others choose to use them instead of conventional medical care. Taking supplements instead of seeking professional medical treatment for influenza could be harmful to your health. It could greatly increase your risk of severe complications, such as pneumonia. It is important to talk to a healthcare professional if you are thinking about taking nutritional supplements.

Side effects

Some dietary supplements can cause adverse gastrointestinal symptoms or interact poorly with standard cold and flu medications. Certain highly concentrated herbal supplements can even interfere with how well prescription antiviral drugs work. Antioxidant supplements, such as selenium and vitamins A, C, and E, change how cells handle oxidative stress. Because of this, some doctors think taking high doses could disrupt the body's natural immune mechanisms used to clear viruses. Get advice from your doctor or pharmacist if you want to take supplements and are taking any kind of prescription medication.

Research on dietary supplements and influenza

There is no strong evidence that any dietary supplement can prevent or cure influenza. However, there is evidence that a balanced diet with lots of fruits and vegetables can support a healthy immune system overall. Some research has looked at whether certain vitamins can help prevent respiratory infections in certain groups of people. Studies investigating high-dose vitamin D and vitamin C supplements found that they did not significantly reduce the incidence or duration of influenza infections in well-nourished individuals.

Risks of Unregulated Ingredients

Another significant concern with using dietary supplements for influenza is the lack of strict market regulation. Unlike prescription antiviral medications, which must pass rigorous safety and purity testing before reaching consumers, dietary supplements do not face the same strict oversight. This means the actual concentration of active ingredients can vary drastically from what is listed on the bottle, or the product may contain unlisted fillers and contaminants. For an individual already weakened by a severe influenza infection, introducing these unverified, highly concentrated botanicals can cause unexpected liver stress, allergic reactions, or strain on the kidneys, ultimately complicating the recovery process.

Organizations that conduct systematic reviews analyze the published results of all available studies on a specific issue to draw conclusions. A major scientific review looked closely at the essential mineral selenium to see if selenium supplements could reduce the risk of viral respiratory infections. After evaluating all the data, researchers found that selenium supplementation does not reduce the risk of contracting influenza. Some of the long-term studies even raised safety concerns, reporting adverse metabolic effects in people who took excessive selenium supplements over an extended period.

# P8

**Natural Supplements for Influenza**

Certain dietary supplements may help reduce the risk of influenza or support the body during recovery from viral infections. Supplements can contain herbal extracts or vitamins and minerals. In this article, we discuss some of the dietary supplements that may help prevent influenza or assist in recovery during illness.

A study found some evidence that the anti-inflammatory properties of omega-3s may lower a person’s risk of developing viral respiratory infections. Dietary supplements that contain omega-3 fatty acids are widely available in health stores and pharmacies in a variety of different forms, including:

- cod liver oil
- fish oil
- krill oil
- algal oil, which comes from algae and is suitable for vegetarians and vegans

A person can also purchase omega-3 supplements online. The National Institutes of Health (NIH) recommend a daily intake of 1.1–1.6 grams (g) of omega-3 fatty acids for people aged 14 years and upwards.

Green tea is a popular drink that is rich in compounds called polyphenols, which have anti-inflammatory and antiviral properties. Supplements containing extracts from green tea and its main active component, epigallocatechin-3-gallate (EGCG), may help in the fight against influenza. According to a review, EGCG and green tea extracts may help prevent or delay viral replication, reducing the severity of flu symptoms. Drinking one or more cups of green tea each day is a simple way to enjoy its potential health benefits. Green tea extracts are readily available as dietary supplements, and people can purchase many of these products online as well.

Garlic and onions belong to the Allium genus of plants. According to a research review, eating more of these plants may help lower the risk of respiratory infections, particularly in the upper respiratory tract. A study found that garlic extract blocks the growth and spread of the influenza virus in test-tube experiments and animal models.

Vitamin C is an essential micronutrient and a powerful antioxidant that supports various cellular functions of the immune system. According to a comprehensive clinical review, regular supplementation with vitamin C can help prevent seasonal respiratory infections and significantly reduce the duration and severity of influenza symptoms once they begin. Vitamin C actively concentrates inside white blood cells, boosting their ability to track down and destroy viral particles. While citrus fruits and bell peppers are excellent food sources, vitamin C supplements are widely accessible in tablets, effervescent powders, and chewable forms.

According to health organizations, research indicates that vitamin D may strengthen the immune system and prevent the development of severe respiratory tract infections. Some studies in humans suggest that higher intakes of vitamin D may lower the risk of contracting influenza during peak winter months. Health guidelines recommend a daily intake of 600 international units (IU) or 15 micrograms (mcg) of vitamin D for most people to maintain optimal immune function.

# C8*

**Vitamins and Influenza: Understanding the Risks**

People who consume plenty of foods containing beta-carotene might have a lower risk of certain respiratory complications, such as viral pneumonia related to influenza. However, studies so far have not shown that vitamin A or beta-carotene supplements can help prevent influenza or decrease the chances of severe illness. In fact, studies show that individuals who regularly smoke or have chronic lung conditions who take high-dose beta-carotene supplements actually face an increased risk of lung irritation and respiratory distress during an active infection.

Individuals with a high intake of vitamin C from fruits and vegetables might have a lower risk of catching seasonal viral infections, such as influenza and respiratory syncytial virus (RSV). However, taking vitamin C supplements, with or without other antioxidants, does not appear to protect people from contracting influenza.

It is also not clear whether taking high doses of vitamin C is useful as a treatment once you are already sick with influenza. The effects of vitamin C seem to depend entirely on how it is administered to the patient. Oral doses of vitamin C cannot increase blood levels of vitamin C nearly as high as intravenous doses given through injections. A few studies in animals and test tubes indicate that extremely high levels of vitamin C in the blood might inhibit viral replication, but these studies have not been successfully replicated in clinical trials on humans. More research is needed to determine if high-dose intravenous vitamin C actually helps treat influenza in patients.

Dietary supplements with vitamin C and other antioxidants could interact poorly with standard prescription antiviral medications for influenza. People who are being treated with target medical therapies for seasonal illness should talk to their healthcare provider before taking vitamin C or other antioxidant supplements, especially in high doses.

High levels of vitamin D in the blood have been associated with unexpected metabolic issues in certain patients. At this moment, it is too early to say definitively whether low levels of vitamin D significantly increase the risk of catching influenza, or if higher levels protect the body or even increase health risks for some people.

Most research indicates that vitamin E does not help prevent influenza and can be harmful in some cases. High doses of vitamin E have not consistently reduced the risk or duration of respiratory illness in clinical studies. For example, a large study found that taking vitamin E supplements (400 IU/day) for an extended period actually disrupted normal immune cell signaling in men. Two studies tracking middle-aged adults for several years found that supplemental vitamin E (averaging 300–400 IU/day) did not protect them from any seasonal viral infections. Dietary supplements with vitamin E and other antioxidants could disrupt the body's natural inflammatory response needed to clear a virus. People managing active illness should discuss with their doctor or pharmacist before taking vitamin E or other antioxidant supplements, especially in high doses.

# P9

**Best Supplements for Anxiety**

Taking supplements and vitamins for anxiety relief can address the three most important biological factors that contribute to anxiety and panic attacks: serotonin deficiency, low vitamin B6 levels and low iron levels. When combined adequately and taken in sufficient dose, supplements can correct many of these biological factors and provide relief from many of the most severe symptoms of anxiety.

Some of the best supplements and vitamins for anxiety include:

1. GABA

Gamma-aminobutyric acid (GABA) is an amino acid and neurotransmitter located in the brain that is crucial to serotonin production. Because serotonin is one of the nervous system’s most powerful, “feel-good” neurotransmitters, GABA plays a significant role in mood regulation and relaxation.

How to use it: While many vitamins improve anxiety by affecting GABA levels in the brain, GABA can also be consumed directly through supplements to reduce anxiety symptoms.

2. Passionflower

Passionflower is a calming herb commonly used as a household treatment for anxiety. Its been shown to promote positive moods, improve sleep quality and alleviate nervousness.

How to use it: Passionflower can be consumed as an extract and tablet, or added into teas and tinctures.

3. Valerian Root

Valerian root has been used for a variety of medicinal purposes since the time of ancient Greece. While valerian root is commonly known as a sleep aid, this herb can also be helpful for reducing anxiety. Once ingested, valeric acids found within the herb convert to calming, “feel-good” neurotransmitters in the body, regulating stress and relaxing the body and mind.

How to use it: Valerian root extract is available in both capsule and liquid form. It is also available as a tea.

4. Licorice Root

People enjoy licorice root for its sweet taste and it’s traditionally used in many candies and beverages. However, this herb also carries many health benefits for people with anxiety because of the effects it has on the adrenal glands. Within the body, the adrenal glands produce the stress hormones adrenaline and cortisol. Licorice helps regulate the production of these hormones, buffeting the body’s defenses against stress and reducing anxiety symptoms. Licorice root can also soothe gastrointestinal upset, which is common in many people with anxiety.

How to use it: Most modern-day drinks and candies that claim to contain licorice only contain licorice flavoring, which doesn’t provide the same nutritional benefits as licorice root. It’s best to consume licorice in an extracted, purified form.

5. Ashwagandha

For centuries, Ashwagandha has been used in Ayurvedic medicine to combat the effects of aging, improve energy and reduce anxiety. In natural medicine, the root is considered to be an “adaptogen,” or a compound that helps regulate the body’s natural processes and promote overall wellness and health. Today, many people use Ashwagandha to improve mood and reduce anxiety symptoms.

How to use it: While the benefits of Ashwagandha are gained by eating the fruit, seeds and shoots of the plant it is derived from, Ashwagandha is most commonly consumed in capsule form.

# C9

**Is there an effective herbal treatment for anxiety?**

Several herbal remedies have been studied as treatments for anxiety, but more research is needed to understand the risks and benefits. Here’s what we know — and what we don’t:

• Kava

Kava seemed to be a promising treatment for anxiety, but reports of serious liver damage — even with short-term use — led the U.S. Food and Drug Administration (FDA) to issue warnings about the use of dietary supplements containing kava. While these initial reports of liver toxicity have been questioned, exercise extra caution and involve your doctor in your decision if you’re considering using kava-containing products.

• Passionflower

A few small clinical studies suggest that passionflower may help with anxiety. In many commercial products, passionflower is combined with other herbs, making it difficult to distinguish the unique qualities of each herb. Passion flower is generally considered safe when taken as directed, but some studies have found that it can cause drowsiness, dizziness, and confusion.

• Valerian

In some studies, people who used valerian reported less anxiety and stress. In other studies, people reported no benefits. Valerian is generally considered safe at recommended doses, but since long-term safety studies are lacking, don’t take it for more than a few weeks at a time unless your doctor approves. It may cause some side effects, such as headaches, dizziness, and drowsiness.

• Chamomile

Limited data shows that short-term use of chamomile is generally considered safe and may be effective in reducing symptoms of anxiety. But chamomile may increase the risk of bleeding when used with blood-thinning medications. Using chamomile may cause allergic reactions in some people who are sensitive to the family of plants that includes chamomile. Other members of this family include ragweed, marigolds, daisies, and chrysanthemums.

• Lavender

Some evidence suggests that oral lavender or lavender aromatherapy may reduce anxiety; however, the evidence is preliminary and limited. Oral lavender may cause constipation and headaches. It may also increase appetite, increase the sedative effect of other medications and supplements, and cause low blood pressure.

• Lemon balm

Preliminary research shows that lemon balm may reduce some symptoms of anxiety, such as nervousness and excitability. Lemon balm is generally well-tolerated and considered safe for short-term use, but it may cause nausea and abdominal pain.

Herbal supplements are not monitored by the FDA in the same way that medications are. If they were, none of the above would pass the test. Furthermore, despite improved quality control regulations in place since 2010, the quality of some supplements can still be an issue. Remember, natural doesn’t always mean safe. Some herbal supplements taken for anxiety can make you drowsy, so they may not be safe to take while driving or performing hazardous tasks. Your doctor can help you understand the possible risks and benefits if you choose to try an herbal supplement.

If your anxiety is interfering with your daily activities, talk to your doctor. More severe forms of anxiety generally require medical treatment or psychological counseling (psychotherapy) for symptoms to improve.

# P10

**Natural Supplements to aid with Anxiety**

It’s a well-established scientific fact that what you eat can dramatically alter your mood . Nutritional deficiencies can exacerbate mental health disorders, while a nutritionally complete diet can help alleviate symptoms. Certain natural vitamins - or vitamins obtained by consuming whole foods - are thought to have particularly positive effects on anxiety. While most natural vitamins also exist in supplement form, they’re more effectively absorbed by the body when ingested by eating whole foods.
Some of the most common natural vitamins for anxiety include:

1. Omega-3 Fatty Acids

Fish oils contain omega-3 fatty acids which make up the basic building blocks of the brain and nervous system. These acids are essential for cognitive functioning and have been proven to improve symptoms of depression, which is often closely linked with anxiety disorders.

Foods that have them: These brain-boosting amino acids are found in a wide variety of fish species, including salmon, tuna, mackerel, lake trout, herring, sardines and anchovies.

2. Probiotics

Probiotics are microorganisms known for their benefits to digestive health. However, recent researcg  has revealed that probiotics can also have a profound impact on mental health. A healthy balance of bacteria in the body can boost the body’s ability to cope with stress, improve overall mental health and bolster cognitive functioning.

Foods that have them: Probiotics are found in a wide variety of foods and drinks, particularly those created through fermentation. Some of the most common sources include sauerkraut, yogurt, kimchi, tempeh, kombucha, miso and pickles.

3. B Vitamins

B vitamins are vital to healthy nervous system functioning. As such, they play a key role in various aspects of mental health, including attention, energy and cognition. They can also have a significant impact on two key aspects of anxiety symptoms: stress management and mood. Because of these benefits, many people incorporate B vitamins into their diet for anxiety.

Foods that have it: While B-complex supplements contain a broad range of essential B vitamins, these key nutrients are also found in a wide variety of foods, including wild salmon, shrimp, tuna, halibut, yogurt, eggs, cheese, lamb, venison, turkey, grass-fed beef, carrots and green, leafy vegetables.

4. L-Theanine

L-theanine is an amino acid that can improve focus, reduce stress and promote relaxation. Research has shown its ability to produce positive effects on mood in humans, and a 2018 study showed that it had demonstrable anti-anxiety benefits in rats.

Foods that have it: L-theanine can only be found in a few foods and drinks, including black tea, green tea and bay bolete mushrooms.

Vitamins for Panic Attacks

Currently, research on the connection between panic attacks and vitamin deficiencies is limited. However, because panic attacks are often a symptom of an anxiety disorder called panic disorder, incorporating any of the above supplements and vitamins into your diet may help you better manage anxiety that can trigger panic attacks.

# C10*

**Herbal and Dietary Supplements for the Treatment of Anxiety Disorders?**

HAWTHORN AND CALIFORNIA POPPY

A single French study exists of a combination product called Sympathyl, which contains 20 mg California poppy, 75 mg hawthorn, and 75 mg elemental magnesium. According to the study, Sympathyl had a very small but positive effect on anxiety. No clinical trials suggest that any of the individual components reduce anxiety in patients with anxiety disorders.

VALERIAN

Although valerian is often cited as having anxiolytic effects and has been used for centuries by herbalists/physicians to treat nervousness, there are only two small trials involving valerian, neither of which produced clear indications of effectiveness. Thus, at the present time, there is no clinical evidence of an anxiolytic effect of valerian when compared with placebo in patients with anxiety disorder.

PASSIONFLOWER

A single randomized double-blind trial compared 45 drops of passionflower tincture per day to 30 mg per day of oxazepam (Serax; brand no longer available in the United States) for 30 days. Investigators noted a marked reduction in anxiety score in both groups, but without a placebo group it was unclear whether other aspects of the milieu could have caused the effects.

ST. JOHN'S WORT

St. John's wort is a popular supplement for treating depression but is much less popular for treating anxiety disorders. Studies specifically testing the effects of St. John's wort on patients with anxiety are extremely limited

The evidence of positive effects of St. John's wort on anxiety disorders is weak. No placebo-controlled, randomized, double-blind trials have shown St. John's wort to be effective in treating generalized anxiety disorder, post-traumatic stress disorder, obsessive-compulsive disorder (OCD), or phobias. The only effective trial involved patients with somatoform disorder, although the relationship between somatoform disorder and anxiety is complex. Much stronger evidence is needed before St. John's wort should be considered a treatment option for patients with diagnosable anxiety disorders.

The vast majority of neurotransmitter or hormonal precursors that claim to be useful for treating anxiety disorders have no evidence supporting clinical utility. Only 5-hydroxytryptophan appeared to show clinical effectiveness among the precursor preparations.

Although there is some indication that 5-hydroxytryptophan can reduce anxiety symptoms among patients with anxiety disorders, the evidence is weak. Also, it has been known to cause eosinophiliamyalgia syndrome, a significantly dangerous side effect. Therefore, the risk/benefit ratio does not favor physician support of patients choosing this medication because it is “natural.”

The existing data show that the popular supplements have little therapeutic value for anxiety disorders, and their use should be discouraged in favor of more effective treatments. In addition, many preparation sthat might be used by patients to reduce anxiety lack evidence of effectiveness with anxiety disorders.

# P11*

**Using Natural Supplements to Treat Diabetes**

A healthy diet, exercise, and maintaining a healthy weight are the first and sometimes most important parts of treating diabetes. However, when these aren’t enough to keep your blood sugar levels in check, there are a number of herbs and supplements you can try to improve your diabetes. These alternative treatments help control blood sugar levels, reduce insulin resistance, and prevent diabetes-related complications. A number of supplements have shown promise as diabetes treatments. These include the following.

Cinnamon

Chinese medicine has used cinnamon for medicinal purposes for hundreds of years. It has been the subject of numerous studies to determine its effect on blood glucose levels. A 2011 study found that cinnamon, in whole or extract form, helps lower blood glucose levels.

Chromium

Chromium is an essential trace element. It is used in carbohydrate metabolism.

Vitamin B-1

Vitamin B-1 is also known as thiamine. Many people with diabetes are deficient in thiamine. This can contribute to some of the complications of diabetes. Thiamine deficiency has been linked to heart disease and blood vessel damage. Thiamine is water-soluble. It has difficulty getting into cells where it is needed. However, benfotiamine, a supplemental form of thiamine, is fat-soluble. It penetrates cell membranes more easily. Some research suggests that benfotiamine may prevent complications of diabetes.

Alpha-lipoic acid

Alpha-lipoic acid (ALA) is a powerful antioxidant. Studies suggest that it can:

• reduce oxidative stress

• lower fasting blood sugar

• decrease insulin resistance

Watermelon

Watermelon is used to treat diabetes-related conditions in places like Asia, South America, and more. There is a lot of data on its effectiveness as a treatment for diabetes in animal and laboratory studies.

Green tea contains polyphenols, which are antioxidants.

The main antioxidant in green tea is known as epigallocatechin gallate (EGCG). Laboratory studies have suggested that EGCG may have numerous health benefits, including:

• lower risk of cardiovascular disease

• prevention of type 2 diabetes

• improved glucose control

• better insulin activity

Resveratrol

Resveratrol is a chemical found in wine and grapes. In animal model studies, it helps prevent high blood sugar levels. Animal studies have also shown that it can reduce oxidative stress.

Magnesium

Magnesium is an essential nutrient. It helps regulate blood pressure. It also regulates insulin sensitivity. Magnesium supplements may improve insulin sensitivity in diabetics. A diet rich in magnesium may also reduce the risk of diabetes. Researchers have found a link between higher magnesium intake, lower rates of insulin resistance and diabetes.

Overview

As you can see from this list, there are a number of natural supplements that can be used to manage diabetes.

# C11

**Beware of Illegally Marketed Diabetes Supplements**

As the number of people diagnosed with diabetes continues to rise, illegally marketed products that promise to prevent, treat, and even cure diabetes are flooding the market.

The U.S. Food and Drug Administration (FDA) advises consumers not to use such products—for a variety of reasons. For example, they may contain harmful ingredients or be mislabeled as over-the-counter products when they should be marketed as prescription products. Illegally marketed products pose an additional risk if they cause people to delay or stop effective diabetes treatments. Without proper management of the disease, people with diabetes are at greater risk of developing serious health complications.

More than 30 million people in the United States have diabetes, and one in four don’t know they have it, according to the Centers for Disease Control and Prevention. Millions of people have prediabetes, which means they have higher-than-normal blood sugar levels, and they can reduce their risk of developing diabetes by making healthy lifestyle changes, including diet and exercise.

“People with chronic or incurable diseases can feel desperate and become easy targets. Counterfeit diabetes products are particularly concerning because there are effective drug options available to help manage this serious disease, rather than exposing patients to unproven and unreasonably risky products,” said Jason Humbert, a captain in the U.S. Public Health Service, which is part of the FDA’s Office of Regulatory Affairs. “Failure to follow well-established treatment plans can lead to amputations, kidney disease, blindness, and death, among other things.”

The FDA has issued warning letters to various companies that market diabetes products in violation of federal law. These products have been marketed as dietary supplements; alternative medicines, such as Ayurvedic; and homeopathic products. Some of the companies also promoted the same unapproved drugs for other serious conditions, including cancer, sexually transmitted diseases, and macular degeneration.

FDA lab tests have found that “all-natural” diabetes products contain undeclared active ingredients found in approved prescription drugs intended to treat diabetes. Undeclared active ingredients can cause serious harm. If consumers and their healthcare professionals don’t know the actual active ingredients in the products they’re taking, these products can interact in dangerous ways with other medications. One potential complication: Patients may end up taking a higher combined dose of diabetes medications than they intended. This can cause a significant and unsafe drop in blood sugar, a condition known as hypoglycemia. "Products that promise an easy fix may be appealing, but consumers are gambling with their health. Overall, diabetes is a chronic disease, but it is manageable. And people can reduce their risk of developing complications by following treatments prescribed by health professionals, carefully monitoring their blood sugar levels, and adhering to a proper diet and exercise program," Humbert said.

# P12

**Common Diabetes Dietary Supplements**

Bitter melon

Intended use. Bitter melon (Momordica charantia) is a green fruit related to cantaloupe, honeydew, casaba, and muskmelon. Found in tropical climates, bitter melon (also called bitter gourd or bitter cucumber) is used mainly in India, Asia, South America, and Africa, both in cooking and as a medicine. The fruit and seeds of the bitter melon are believed to lower glucose levels; possible modes of action include increased glucose uptake by tissues, increased muscle and liver glycogen synthesis, and enhanced glucose oxidation.

Researchers have isolated four compounds from bitter melon that activate an enzyme called AMPK. AMPK regulates fuel metabolism and promotes glucose uptake in a manner similar to exercise. Only a small handful of studies have examined the effect of bitter melon (in varying forms) on diabetes; glucose and A1C levels decreased, although not significantly.

Potential side effects. Bitter melon eaten as a vegetable is likely to be safe. People allergic to melons may experience an allergic reaction to bitter melon; symptoms include rash, itching, and shortness of breath. The main side effect of bitter melon, however, is gastrointestinal distress. Other adverse effects include headache, hemolytic anemia, and hypoglycemia if ingested with medications that lower blood glucose.

Pregnant women should avoid bitter melon due to the risk of possible birth defects and miscarriage. In addition to enhancing the effect of glucose-lowering medication, bitter melon may interact with lipid-lowering drugs, immune system suppressants, and chemotherapy medications.

Chromium supplementation

Intended use. Chromium is an essential trace mineral that is needed for glucose metabolism. It works by enhancing the effect of insulin. Chromium, usually in the form of chromium picolinate, is a popular supplement among people with diabetes and those who are interested in losing weight. Its effect on glucose levels has been researched, with mixed results. Chromium is found primarily in two forms: trivalent, a biologically active form found in food, and hexavalent, which is toxic and a result of industrial pollution.

The effects of chromium supplementation in people with diabetes have been mixed. Although a meta-analysis of the effects of chromium supplements on A1C, glucose, and insulin levels showed little effect among those with and without diabetes, other studies have shown some benefit on these same markers in subjects who were chromium deficient. Other studies have shown little, if any, benefit of chromium supplements on diabetes.

Potential side effects. Excessively high intakes of chromium may cause renal and liver failure, thrombocytopenia, hemolysis, skin reactions, and mood disturbances. Chromium may interact with a number of medications, including anatacids, H2 blockers, proton pump inhibitors, β-blockers, corticosteroids, nonsteroidal anti-inflammatory drugs, and nicotinic acid. In addition, patients who take insulin or insulin secretagogues and chromium may have an added risk of hypoglycemia.

# C12*

**Do Dietary Supplements Get a Passing Grade in Managing Diabetes?**

Can taking a pill lower your A1C, reverse insulin resistance, or manage your blood sugar? Never has a single question launched so many health newsletter articles—not to mention so many online and pharmacy purchases. "My patients ask a lot about blood sugar supplements, and I try to point them toward whatever evidence we actually have," says Dr. JoAnn Manson, a professor of medicine at Harvard Medical School and a chief of preventive medicine who has led major, large-scale clinical trials on nutrition.

Dr. Manson’s list of supplements that people frequently ask about includes minerals (chromium, magnesium, zinc), antioxidants (alpha-lipoic acid, vitamin C and E), herbal supplements (cinnamon, berberine, fenugreek), and nutraceuticals (apple cider vinegar pills). For now, you can cross all of these products off your shopping list due to a lack of rigorous clinical evidence. "We absorb vitamins and minerals much better when they come from real food," says Dr. Manson, noting that for most of these concentrated products, there are simply no large clinical trials proving they are effective or safe for metabolic health.

But there is a bright spot in the approach to keeping your metabolism healthy as you age. It comes in the form of healthy eating and regular exercise. "There is absolutely no substitute for a well-balanced diet," says Dr. Manson. Robust data from multiple long-term trials show that establishing a nutrient-dense, carbohydrate-conscious lifestyle pattern can successfully prevent the onset of type 2 diabetes and slow the progression of existing blood sugar challenges much more effectively than a pill.

Lack of Evidence

If we have reliable scientific information about supplements for maintaining insulin sensitivity and blood sugar control, it must come from randomized, double-blind, placebo-controlled clinical trials. In these high-standard studies, a growing number of trials have failed to identify a consistent benefit for blood sugar management from popular options like cinnamon or chromium. For example, major systematic reviews analyzing the data found that while chromium may show slight improvements in rare cases of severe clinical deficiency, it does not reliably lower long-term fasting blood glucose or A1C levels for the average person diagnosed with type 2 diabetes.

In addition, taking highly concentrated versions of these elements carries hidden medical risks. Supplements like berberine, high-dose chromium, and alpha-lipoic acid can cause unexpected gastrointestinal distress or place unnecessary stress on your liver and kidneys. Furthermore, combining these unregulated supplements with standard prescription diabetes medications, such as metformin or insulin, can cause blood sugar levels to drop unpredictably low, creating a dangerous state of hypoglycemia.

Supplement Buyers: Beware

*Caveat emptor*, the Latin phrase for "buyer beware," should be your guide when it comes to considering over-the-counter supplements for diabetes. Because of standard regulatory frameworks, dietary supplements are not classified as drugs. Therefore, they do not have to pass the rigorous approval processes required by agencies like the FDA to ensure they are safe and effective before they hit the shelves. This means many of these products claim to "support" or "help maintain" healthy blood glucose purely because of a marketing loophole—not because researchers have verified those claims to be true.

# S1

**Hubble Telescope captured in detail the explosion of a star 500 times larger than the Sun, from 11 billion years ago**

Around 11.5 billion years ago, a distant star that was about 530 times larger than the Sun in our solar system died in a cataclysmic explosion. The blast hurled its outer gaseous layers into surrounding space, forming a supernova that astronomers have now been able to observe in exceptional detail.

American researchers announced that NASA’s Hubble Space Telescope managed to capture three distinct images that show an eight‑day interval beginning just a few hours after the detonation of this massive star. It is an even more remarkable achievement given how long ago and how far away the event took place. The images were identified in a review of Hubble’s archival data from 2010, according to astronomer Wenlei Chen, a postdoctoral researcher at the University of Minnesota and lead author of the study published in the journal Nature.

For the first time, a rapidly cooling supernova was observed within a single set of images, providing the first detailed view of such an explosion from such an early period in the history of the Universe, when it was less than a fifth of its current age. “The supernova expands and cools, so its color evolves from intense blue to deep red,” explained Patrick Kelly, a professor of astronomy at the University of Minnesota and coauthor of the study.

The dying star, belonging to a class called red supergiants, was located in a dwarf galaxy and exploded at the end of its relatively short life span. “Red supergiants are bright, massive, and very large, but they are much cooler than most other massive stars, which is why they appear red,” said Wenlei Chen.

“After a red supergiant exhausts the nuclear fusion fuel in its core, the core collapses, and the supernova explosion disperses the star’s outer layers – the hydrogen envelope,” Chen added.

The first image, which captures the event around six hours after the initial explosion, shows the extremely hot and relatively compact onset of the blast – roughly 180,000 degrees Fahrenheit (100,000 Kelvin / about 99,725 degrees Celsius). The second image was taken two days later, and the third about six days after the explosion. In these last two images, the gaseous material ejected from the star can be seen expanding outward. In the second image, the explosion is about five times cooler than in the first, and in the third, around ten times cooler than in the first. The remnants of the exploded star most likely formed an incredibly dense object known as a neutron star, according to Wenlei Chen.

# S2

**NASA launched an experimental shield that will help humans reach Mars**

On Thursday, NASA launched a satellite designed to improve weather forecasting, together with an experimental heat shield intended to help humans one day reach Mars.

The two missions were sent into space on a United Launch Alliance Atlas V rocket from California. Atmospheric satellites have been launched since the 1960s, but what is new about this launch is the low‑Earth‑orbit flight test of an inflatable decelerator – LOFTID.

The mission is designed to test inflatable heat‑shield technology needed for landing on Mars, where a human mission is also being considered, as well as for potential missions to Venus or Saturn’s moon Titan. LOFTID could also be used when returning heavy payloads to Earth. Sending robotic explorers or humans to other planets that have atmospheres is difficult because traditional rigid heat shields are limited by the size of the rocket. An inflatable aerodynamic shell could solve this problem and allow larger spacecraft to be sent to other worlds.

When a spacecraft enters a planet’s atmosphere, it encounters aerodynamic forces that help slow it down. On Mars, where the atmosphere is only about 1% as dense as Earth’s, additional help is needed to create enough drag to slow and safely land a spacecraft. That is why NASA engineers believe that a large aerodynamic shell that can be deployed like a parachute, such as LOFTID, which inflates and is protected by a flexible heat shield, could provide the necessary braking while traveling through the Martian atmosphere.

The shield is designed to generate more drag in the upper atmosphere, helping the spacecraft slow down earlier, which also reduces some of the extreme heating it would otherwise experience. The LOFTID prototype launched into space has a diameter of about 6 meters.

At present, NASA can land around one metric ton on the Martian surface, roughly the mass of the car‑sized Perseverance rover. But a shield like LOFTID could make it possible to land 20–40 metric tons on the Red Planet.

# S3

**The first space factory is being built: when it will be launched into orbit and what it could produce**

A young European company, Space Cargo Unlimited, plans to build by 2025 a vehicle that will serve as a space factory.

The contract to manufacture REV1, an autonomous uncrewed spacecraft, was awarded to Thales Alenia Space (TAS), a company specialized in pressurized modules, the Luxembourg‑ and France‑based startup announced.

The spacecraft, with a mass of under three tons, will be reusable up to 20 times and will remain in low Earth orbit a few hundred kilometers above the planet for two to three months during each mission.

The design builds on TAS’s experience in this field. TAS is developing the Space Rider vehicle for the European Space Agency (ESA), following the Intermediate eXperimental Vehicle (IXV), which flew successfully in 2015. “We have raised the funds necessary to ensure the development of the vehicle’s first phase,” said Nicolas Gaume, CEO of Space Cargo Unlimited.

The company is betting on a new emerging economy – space manufacturing – which Morgan Stanley estimates could reach a value of 10 billion dollars by 2040. The applications are numerous, from agriculture and pharmacology to new materials, Gaume explained. Space Cargo has already sent 320 grapevine plants into space for 14 months. “Exposure to the space environment led to evolutionary changes that gave these plants naturally increased resistance to climate change,” he said, noting that “gravity is the only parameter of life that has not changed for 4 billion years” and that the absence of gravity leads to “accelerated evolution.”

The company intends to bring to market these more resilient varieties of Cabernet Sauvignon and Merlot in 2024. Space manufacturing could also allow high‑precision polishing of optical lenses without the constraints of gravity and could even provide “perfectly clean rooms” for manufacturing microprocessors, for example.

# S4

**The planets in our solar system where it rains diamonds**

Uranus and Neptune are two of the many mysterious celestial objects in the Universe that the James Webb Space Telescope will soon begin to explore. On these planets, temperature and pressure conditions are so extreme that carbon atoms can transform into diamonds in their atmospheres.

Astrophysicist Naomi Rowe‑Gurney, who specializes in studying these two planets, explained this process in an interview published on NASA’s website. “I love these ice giants, mostly because they haven’t been studied very much. I initially wanted to do my PhD on these two planets because there were so many unanswered questions. We simply do not even know how they formed and why they are the way they are. We don’t even know if it is correct to call them ice giants, because who knows what is inside?” she said.

Rowe‑Gurney notes that Uranus and Neptune are blue because they have high levels of methane in their atmospheres. “I study their middle atmosphere, which is the stratosphere and the upper troposphere. It’s at about one bar, which is roughly the same pressure that we have here on Earth in the air,” she said. “We see the Sun interacting with methane in the atmosphere and breaking it apart into many different hydrocarbons. Many of these molecules are made up of hydrogen and carbon and have lots of fancy names like diacetylene, acetylene, and methylacetylene. And we still don’t really know everything, such as the full composition of everything that is there, and we’re still finding new things all the time. That is why the James Webb Telescope is so exciting, because we will be able to see many more things on these two planets.”

The astrophysicist explained that methane contains carbon, which can be crushed by the immense pressures deeper in the atmosphere, forming diamonds. Inside the planet, where everything becomes very hot and very dense, these diamonds form and accumulate, then become heavier and heavier. “That means it rains in the atmosphere. But it is not rain like we see here, because these pressures are extreme, and we will never be able to reach those depths. So even if those diamonds exist, we would never be able to collect them,” she explained.

# S5

**Hubble detected a dead star that is devouring its own planetary system – an act of “cosmic cannibalism”**

The Hubble Space Telescope has observed a white dwarf star that is devouring rocky and icy material from its own planetary system.

This suggests that water and other volatile substances may be common in the outer regions of planetary systems. Astronomers used archival data from Hubble and other observatories to analyze the spectral properties of the white dwarf G238‑44. The elements detected show that the dead star is swallowing debris from both the inner and outer parts of its system. It is a case of “cosmic cannibalism,” say the authors of the study, published on the Hubble Telescope website.

“We have never seen both types of objects accreting onto a white dwarf at the same time,” said Ted Johnson, lead author of the research and a recent graduate of the University of California, Los Angeles. “By studying these white dwarfs, we hope to better understand the planetary systems that are still intact,” he explained. G238‑44 was once a Sun‑like star that has lost its outer layers and no longer burns fuel through nuclear fusion.

The discovery that the stellar remnant is capturing material simultaneously from its asteroid belt and from Kuiper‑belt‑like regions at the edge of the system, including icy bodies, is significant because it suggests that a “water reservoir” may be a common feature in the outer regions of planetary systems.

“Life as we know it requires a rocky planet covered with a variety of elements such as carbon, nitrogen, and oxygen,” said Benjamin Zuckerman, professor emeritus in the Division of Astronomy and Astrophysics at UCLA and coauthor of the study. “The abundance of elements we see on this white dwarf seems to require both a rocky body and one rich in volatiles. This is the first example we have found in hundreds of white dwarfs we have examined,” he explained.

When a star like the Sun expands into a red giant at the end of its life, it loses mass as it sheds its outer layers. A consequence of this process can be the gravitational scattering of small objects such as asteroids, comets, and moons toward the system’s giant planets. After being perturbed in this way, surviving objects can be thrown onto very eccentric orbits.

“After the red giant phase, the remaining white dwarf is compact – no larger than Earth. Planets and smaller bodies can end up coming very close to the star and experience intense tidal forces that tear them apart, creating a disk of gas and dust that eventually falls onto the surface of the white dwarf,” Johnson explained.

# S6

**The most toxic place on Earth helps scientists understand how life could have existed on Mars**

Near the summit of the Poás volcano in Costa Rica lies one of the most acidic lakes on Earth, bright blue and filled with toxic metals. The harsh conditions in Laguna Caliente, where temperatures can fluctuate between 38 degrees Celsius and 90 degrees Celsius, help scientists imagine what life might have been like on the planet Mars.

Frequent phreatic eruptions occur when groundwater is heated by volcanic activity, releasing blasts of ash, rock, and steam. Yet microbes have found a way to live in this environment, one of the most hostile on our planet, according to multiple studies of the lake and new research published in the journal Frontiers in Astronomy and Space Science. Although the diversity of life in this lake is low, it has managed to adapt.

“Our discovery shows that life persists in the most extreme environments on Earth,” said study author Justin Wang, a research assistant at the University of Colorado Boulder. “It is hard to imagine anything more hostile to life than an ultra‑acidic volcanic lake with frequent eruptions. The low biodiversity, together with the many adaptations and metabolisms we found in our sample, suggests that the lake hosts microbes that are very specialized for this type of environment,” Wang said.

This environment could serve as an analogue for how life might have existed on Mars billions of years ago and could point scientists to new places to search for signs of ancient life on the Red Planet, according to the researchers. Poás volcano, located in the middle of Costa Rica’s tropical forest, last erupted in 2017 and 2019. The area around the volcano is devoid of life because of the toxic gases it releases. Wang and his collaborators hiked to the volcano in November, a month after the crater lake had reformed. Portions of the lake were boiling, and volcanic vents called fumaroles were belching hot sulfurous gases.

“We went to Poás volcano more than a year after the eruptions and only a month after the lake had reformed. The lake itself is turbulent and dynamic. As you get closer, you can smell the strong odor of sulfur, which clung to the clothes we wore that day and is still there. Even worse is the smell of hydrochloric acid, which tastes sour in the air and stings your eyes,” Wang said.

Around the lake there are pools of boiling, acidic water, and Wang could feel the heat of the volcano through the soles of his boots near the shoreline. The researchers collected samples from the lake, as they had done in 2013 and 2017. In 2013, they discovered that Acidiphilium bacteria live in the lake. These microbes are often found in acidic mine drainage and hydrothermal systems like Laguna Caliente. Acidiphilium bacteria have multiple genes that allow them to adapt and survive in different environments. Several eruptions occurred before the team returned in 2017. After collecting more samples, the researchers found that there was slightly more biodiversity among the lake’s bacteria than expected.

In addition, DNA sequencing showed that Acidiphilium bacteria had developed ways to convert elements such as sulfur, iron, and arsenic to generate the energy needed for survival. The genetic adaptations identified by Wang and colleagues suggest that life could have survived in hydrothermal environments on Mars in the same way that it does in some of the most extreme places on Earth.

# S7

**A white dwarf suddenly flared and faded again in 30 minutes, under astronomers’ eyes**

A space probe searching for new planets recorded a white dwarf star that suddenly became brighter and then faded back again in just 30 minutes. It is the first time astronomers have seen a white dwarf change its brightness so quickly. This observation may help them understand how matter accumulates onto various types of cosmic objects.

White dwarfs are the remnants of much larger stars, similar in size to our Sun, that have burned through all the hydrogen that once fueled them. As such, astronomers say that studying systems with white dwarfs helps us learn what the distant future of our own solar system will look like, when the Sun will have run out of hydrogen in about 5 billion years.

The discovery was made using NASA’s Transiting Exoplanet Survey Satellite (TESS), whose primary mission is to search for Earth‑sized worlds relatively close to our planet in a broader effort to understand how common life might be in the Universe. In this case, TESS observed fluctuating brightness in a star called TW Pictoris, located about 1,400 light‑years from Earth.

The brightness changes occurred because the white dwarf is drawing material from a nearby companion star, in a process known as accretion. As the white dwarf “feeds” on its companion, it becomes brighter. The white dwarf star then lost its brightness in just 30 minutes, much faster than other white dwarfs that fade over the course of days or even months. Astronomers cannot yet fully explain this phenomenon, because the flow of material in the white dwarf’s accretion disk should be relatively constant, but they suspect that fluctuations in the star’s magnetic field may be disrupting the flow of matter.

“The brightness of a white dwarf is affected by the amount of surrounding material it is feeding on, so researchers say that something is interfering with its ‘supply’ of matter,” explained Durham University, which led the research, in a statement.

# S8

**Why we will not be able to travel faster than the speed of light. Astrophysicist: The Universe has a speed limit**

The Universe has a speed limit, and it seems we cannot get around it, says astrophysicist Adam Becker. He explains why we will not be able to travel faster than the speed of light.

“I grew up with Star Trek. Back then I thought it was a documentary about the future and that we would find ways to travel faster than light. Then I learned more physics and I was very upset,” the astrophysicist says.

“If you want to reach the speed of light, you need force. This is something Isaac Newton talked about a few hundred years ago. He said that an object in motion will remain in motion, and an object at rest will remain at rest unless acted upon by an external force. And an external force with a source of energy is required to change the speed. The faster you want to go, the more force you need,” Becker explained.

“And relativity says that the closer you are to the speed of light, the much more energy you need. And to reach the speed of light with a mass greater than zero, you would need an infinite amount of energy. And to exceed the speed of light, it is impossible to obtain enough energy,” he added.

On the other hand, if something existed that could travel faster than light, it would be impossible for it to slow down, because it would need an infinite amount of energy to reduce its speed, Becker says. Moreover, if something traveled faster than light, it would emit radiation.

“It would be like a sonic boom. When airplanes exceed the speed of sound, you hear a sonic boom. The same thing would happen when exceeding the speed of light: you would have a visual boom. And something that traveled faster than light would lose energy, which would make it move even faster, losing even more energy. It would be an infinite loop of huge amounts of radiation being released by that object going faster than light,” Becker explains.

Furthermore, if this were possible, we could send signals back in time, he adds.

On the other hand, the astrophysicist says that there is no theoretical limit to the speed we can reach, and that we could get very close to the speed of light, which would enable humanity to travel through space. “But if you made such a journey, by the time you came back, billions of years would have passed on Earth,” Becker adds.

# S9

**Scientists have managed to find out what the inside of Mars looks like**

Scientists say that they have managed, based on absolute measurements, to determine what the interior of the planet Mars looks like. The data took them by surprise.

The numbers come from NASA’s InSight lander, which has been detecting quakes on the Red Planet since early 2019. The data reveal that the average thickness of the Martian crust is between 24 and 72 kilometers, thinner than scientists had expected. But the key finding is the size of the planet’s core. Its radius is 1,830 kilometers, more than half the planet’s total radius and larger than researchers had estimated.

It is the first time that science has directly measured the internal layers of a planet other than Earth. Similar measurements have been made for the Moon, but Mars, with a total radius of 3,390 kilometers, is much larger. This information allows researchers to better understand the formation and evolution of different planetary bodies.

The InSight lander obtained these results in the same way seismologists study Earth’s interior, by tracking seismic waves from quakes. These events release waves of energy. Changes in the trajectory and speed of the waves reveal the nature of the rock materials they pass through. NASA’s seismometer system on InSight has recorded hundreds of quakes, and a few of those that occurred in the last two years had the right properties to “image” the interior of Mars.

Based on these data, scientists concluded that the planet’s rigid outer shell – the crust – is 20 or 39 kilometers thick beneath the lander (depending on the exact layering model).

Extrapolating these data to the known surface geology of the rest of the planet suggests an average crustal thickness between 24 and 72 kilometers. By contrast, the average thickness of Earth’s crust is 15–20 kilometers. Only in continental regions such as the Himalayas can it reach 70 kilometers.

Data from Martian quakes show that the core begins at nearly half the distance from the surface to the center, at a depth of about 1,560 kilometers, and that it is in a liquid state.

Most previous estimates pointed to a much smaller core. The research team says that two fascinating consequences arise from the new observations. The first is that the core is much less dense than previously thought and that the iron‑nickel alloy that dominates its composition is probably strongly enriched with lighter elements such as sulfur.

The second consequence concerns the layer between the core and the crust – the mantle. It is thinner than previously supposed and appears to be a simple rock layer without major gradations, unlike Earth’s mantle. Given Mars’s known size, it is very unlikely that the mantle can reach the pressures at which bridgmanite, a mineral that represents more than 90% of Earth’s lower mantle, becomes stable.

On Earth, this rigid mineral envelops the core, slowing convection and heat loss. On Mars, its absence would have led to rapid cooling of the planet. This would initially have allowed strong convection in the metallic core and a dynamo effect that generated a global magnetic field. But that is no longer happening. Today, no global magnetic field can be detected on the planet.

# S10

**Extremely rare phenomenon: a tear‑shaped star and its partner that “devours” it. Astronomers expect a massive supernova**

Astronomers have discovered a rare tear‑shaped star located about 1,500 light‑years from the Sun. Its unusual shape is due to the fact that it has a companion star that is effectively feeding on material from its body. In such situations, both stars eventually explode in a violent supernova visible across the galaxy.

Astronomers are amazed by this partnership. The stellar system, named HD265435, is one of only three known binary systems of this type in the Universe and the closest to Earth. It is expected to explode as a type Ia supernova, according to a study published on July 12 in the journal Nature Astronomy.

This type of explosion occurs when a white dwarf star shares an orbit with a larger, younger star that still has fuel to burn. Small but extremely massive gravitationally, the white dwarf devours this fuel, drawing so much material that the younger star begins to change shape into an ellipse or a tear. The older star gradually grows over millions of years, eventually becoming too massive. Nuclear reactions reignite in its core, the white dwarf’s mass increases, and both stars end up as a glowing smear of gas and dust across the night sky.

Supernovae are relatively easy to observe – one such explosion was visible from Earth for 23 days and nights in the year 1054 CE – but finding stellar systems that will eventually produce type Ia supernovae is much more difficult. This is partly because white dwarfs are very faint and small, according to NASA.

Finding the white dwarf’s companion is not much easier, but because these younger stars are much brighter, they provide important clues, the authors of the new study wrote. One clue is an “ellipsoidal” shape, suggesting that something massive is pulling on its material and distorting it. Another clue is a rapidly pulsing light signature that indicates a binary system in which two stars orbit each other extremely quickly.

Based on observations from NASA’s Transiting Exoplanet Survey Satellite (TESS), researchers discovered that the HD265435 system fits the criteria. Using these details, the team calculated the distance and mass of the bright star, which allowed them to estimate the size and age of the invisible white dwarf. The two stars orbit one another in about 90 minutes, indicating that they are extremely close.

Researchers expect the system to explode in about 70 million years.

# S11

**NASA discovered a galaxy with a “fascinating secret”**

NASA’s Hubble Space Telescope captured an image of a spiral galaxy that hides a supermassive black hole at its center. The galaxy, NGC 3254, is located about 118 million light‑years from Earth.

“NGC 3254 looks like a typical spiral galaxy seen from the side. But it has a fascinating secret hidden in plain sight – it is a Seyfert galaxy. Seyfert galaxies have extraordinarily active nuclei that release as much energy as the rest of the galaxy combined,” NASA explains in a statement.

The black hole at the center of the galaxy, with its immense gravitational power, can draw in gas, dust, stars, and planets. The image was captured by Hubble using both visible and infrared light. Although one might assume that black holes, with their enormous pull, simply devour everything that comes near them, researchers have found that the accumulation of material releases huge amounts of radiation, which in turn emit heat and light. Scientists assembled this image using a composite of light from different parts of the spectrum.

Using Hubble’s WFC3 camera, scientists can observe the Universe in ultraviolet, visible, and infrared light. This galactic phenomenon is relatively unusual, however, because Seyfert galaxies make up only about ten percent of the galaxies in the known Universe.

The image will give scientists the opportunity to look back to a period between 150 million and 1 billion years after the birth of the Universe, which had previously been inaccessible to them.

# S12

**A massive gamma‑ray explosion observed in detail by astronomers: “They occur when a star collapses and becomes a black hole”**

A cosmic explosion of unprecedented proportions that released gamma radiation after a star collapsed under its own weight was directly observed by researchers in Namibia.

The gamma‑ray burst was “captured” by the High‑Energy Stereoscopic System on August 29, 2019, after the Swift and Fermi satellites detected an increase in radiation levels in the constellation Eridanus.

“Gamma‑ray bursts are flashes that can be seen in the sky, emitted at great distances from sources outside the galaxy,” says Sylvia Zhu, a researcher at the German research center Deutsches Elektronen Synchroton.

“These are the largest explosions in the Universe, and they occur when a massive star collapses into a black hole. Part of the released gravitational energy takes the form of a shock wave. The emission is divided into two distinct phases – first, a short phase of a few tens of seconds, followed by a longer one in which the light begins to fade,” she explains.

This type of explosion has never before been observed at such a small distance from Earth – they usually occur around 20 billion light‑years away, but this one took place at only about one billion light‑years. The comparatively much shorter distance meant that researchers were able to see the “colors” of the radiation. The explosion had an energy spectrum of 3.3 tera‑electronvolts, a trillion times more energetic than visible‑light photons. Even three days after the explosion, researchers could still observe its “remnants,” and the findings are extremely important for understanding how radiation works.
