# Supplementary material for: Impact of Repeated Exposure to Polarized Health-Related News on Explicit and Implicit Attitudes Toward Dietary Supplements: Online Experimental Study
Source: JMIR Infodemiology. 2026 Jul 27;6:e88632. doi: 10.2196/88632 (PMC13404937; doi:10.2196/88632)

Multimedia Appendix 2

Table S1. Search terms and pretest quality ratings (N = 15) for candidate article stimuli

| Search Term | PRO M (SD) | CON M (SD) |
| --- | --- | --- |
| Dietary Supplements | 2.87 (.64) | 2.93 (.89) |
| Dietary Supplements for Sleep | 3.03 (.93) | 3.20 (.80) |
| Dietary Supplements for Weight Loss | 2.97 (.67) | 2.93 (.69) |
| Dietary Supplements Influenza | 3.27 (.70) | 3.30 (.80) |
| Dietary Supplements Mental Health | 3.47 (.66) | 3.03 (.74) |
| Dietary Supplements Diabetes | 3.20 (.67) | 3.23 (.64) |
| Dietary Supplements Cancer* | 2.27 (.99)* | 2.90 (.97)* |

Note. During pretesting, participants read each article in a randomized order and rated its perceived quality on a four-star scale. Stimuli marked with an asterisk (*) were removed from the final study to avoid exposing participants to articles on assessment days.

Table S2. Means and Standard Deviations for All Study Variables, Including Engagement and Exposure Measures by Group and Time Point.

|  | PRO (n = 68) | | CON (n = 51) | | MIX (n = 52) | | Control (n = 57) | |
| --- | --- | --- | --- | --- | --- | --- | --- | --- |
|  | *M* | *SD* | *M* | *SD* | *M* | *SD* | *M* | *SD* |
| T0 | | | | | | | | |
| Implicit Attitudes | -.08 | .42 | .00 | .48 | -.12 | .46 | -.17 | .43 |
| Explicit Attitudes | 190.21 | 62.74 | 202.25 | 56.37 | 200.17 | 44.44 | 203.60 | 48.83 |
| Efficiency rating | 61.99 | 24.68 | 66.86 | 20.82 | 67.79 | 16.83 | 67.18 | 20.17 |
| Harmfulness rating | 29.00 | 19.87 | 24.25 | 18.10 | 29.06 | 20.62 | 26.16 | 17.44 |
| Recommendation willingness | 56.22 | 27.11 | 58.65 | 25.43 | 60.44 | 19.42 | 61.58 | 22.79 |
| Time spent reading study articles (s) | 1164.82 | 751.06 | 1205.21 | 1117.17 | 1556.27 | 106.60 | 1339.18 | 856.19 |
| Minutes spent reading health-related news | 24.00 | 22.88 | 54.22 | 166.62 | 26.33 | 35.77 | 56.81 | 120.07 |
| Frequency of reading health-related news | 3.31 | 1.51 | 3.25 | 1.47 | 2.87 | 1.34 | 3.39 | 1.37 |
| Frequency of exposure to positive supplement news | 33.50 | 29.64 | 31.06 | 34.14 | 26.63 | 30.05 | 35.35 | 30.48 |
| Frequency of exposure to negative supplement news | 11.74 | 19.46 | 9.61 | 17.15 | 10.52 | 16.92 | 12.56 | 18.17 |
| Total article ratings | 36.93 | 5.22 | 38.37 | 5.84 | 38.27 | 4.47 | 37.14 | 7.49 |
| T1 | | | | | | | | |
| Implicit Attitudes | -.05 | .43 | -.13 | .47 | -.30 | .47 | -.18 | .45 |
| Explicit Attitudes | 201.31 | 51.03 | 150.80 | 57.67 | 184.92 | 41.35 | 188.12 | 49.91 |
| Efficiency rating | 67.93 | 21.33 | 49.82 | 21.43 | 64.29 | 18.69 | 62.72 | 20.07 |
| Harmfulness rating | 27.69 | 20.65 | 45.35 | 22.19 | 35.02 | 18.06 | 28.77 | 18.48 |
| Recommendation willingness | 60.07 | 22.32 | 45.33 | 23.12 | 54.65 | 21.34 | 53.18 | 23.06 |
| Minutes spent reading health-related news | 26.76 | 25.09 | 39.86 | 49.55 | 34.85 | 43.19 | 58.53 | 99.20 |
| Frequency of exposure to positive supplement news | 25.40 | 24.27 | 21.53 | 25.81 | 20.44 | 25.12 | 27.47 | 26.25 |
| Frequency of exposure to negative supplement news | 14.04 | 19.93 | 27.18 | 27.50 | 12.85 | 18.00 | 14.05 | 18.55 |
| Article ratings | 18.46 | 2.87 | 19.44 | 3.21 | 19.25 | 2.64 | 18.62 | 3.56 |
| T2 | | | | | | | | |
| Implicit Attitudes | -.15 | .41 | -.09 | .39 | -.23 | .41 | -.13 | .40 |
| Explicit Attitudes | 206.15 | 49.22 | 148.06 | 55.75 | 176.40 | 45.61 | 187.54 | 52.23 |
| Efficiency rating | 68.94 | 18.67 | 47.37 | 22.13 | 61.58 | 20.45 | 60.65 | 22.06 |
| Harmfulness rating | 26.01 | 18.36 | 44.08 | 20.93 | 37.62 | 21.13 | 25.98 | 16.56 |
| Recommendation willingness | 62.22 | 20.84 | 43.76 | 23.65 | 51.44 | 23.20 | 51.88 | 24.04 |
| Minutes spent reading health-related news | 25.74 | 21.31 | 30.59 | 34.09 | 39.35 | 53.99 | 50.09 | 84.42 |
| Frequency of exposure to positive supplement news | 24.62 | 24.62 | 16.20 | 20.49 | 22.67 | 23.99 | 28.26 | 27.42 |
| Frequency of exposure to negative supplement news | 16.31 | 23.58 | 25.02 | 26.99 | 14.15 | 19.25 | 12.72 | 16.43 |
| Article ratings | 18.47 | 3.07 | 18.92 | 3.03 | 19.02 | 2.83 | 18.53 | 4.41 |

Sphericity analyses:

Implicit attitudes: Mauchly’s test indicated that the assumption of sphericity was met, *p* = .856.

Explicit attitudes: Mauchly’s test indicated that the assumption of sphericity was violated, *W* = .809, χ²(2) = 45.05, *p* < .001; therefore, degrees of freedom were corrected using the Greenhouse-Geisser estimate (ε = .84).

Figure S1. Moderated mediation models examining the role of baseline explicit and implicit attitudes in the relationship between exposure condition, reading time, and explicit attitude change between T0 and T2


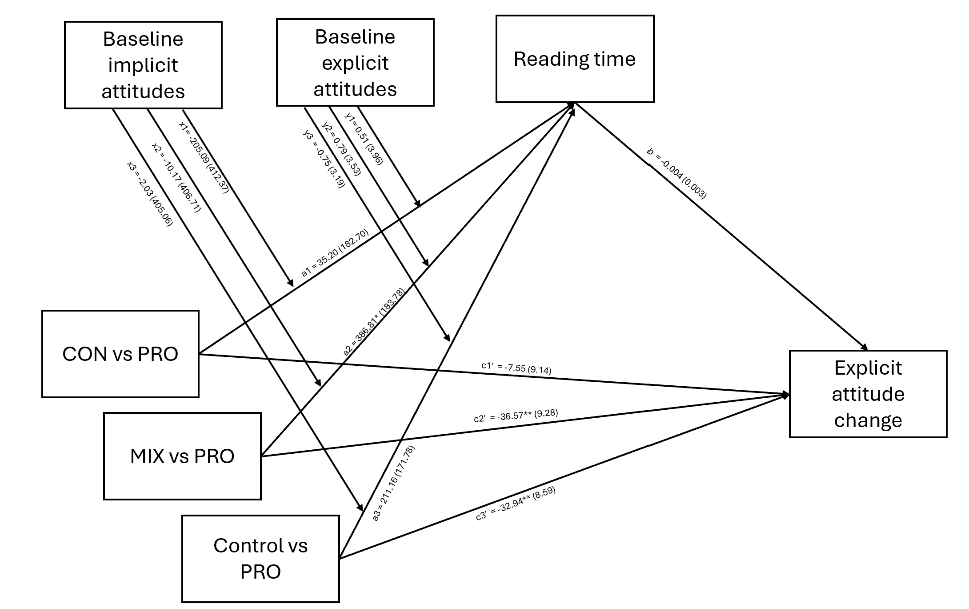

Supplement: Multimedia Appendix 2 [file infodemiology-v6-e88632-s002.docx]
